# Dragon’s Blood Regulates Rac1-WAVE2-Arp2/3 Signaling Pathway to Protect Rat Intestinal Epithelial Barrier Dysfunction Induced by Simulated Microgravity

**DOI:** 10.3390/ijms22052722

**Published:** 2021-03-08

**Authors:** Yujuan Li, Shan Liu, Huayan Liu, Yaoyuan Cui, Yulin Deng

**Affiliations:** School of Life Science, Beijing Institute of Technology, No.5 Zhongguancun South Street, Haidian District, Beijing 100081, China; lynn63333@163.com (S.L.); lhy7881230@163.com (H.L.); cuiyaoyuan7370@163.com (Y.C.); deng@bit.edu.cn (Y.D.)

**Keywords:** Dragon’s blood, simulated microgravity, intestinal epithelial barrier, Rac1-WAVE2-Arp2/3 pathway

## Abstract

Dragon’s Blood is a red resin from Dracaena cochinchinensis (Lour.) S.C. Chen (Yunnan, China). As a traditional Chinese medicinal herb, it has shown protective effects on intestinal disorders. Microgravity could alter intestinal homeostasis. However, the potential herbal drugs for preventing intestine epithelial barrier (IEB) dysfunction under microgravity are not available. This study aimed to investigate the effects of Dragon’s Blood (DB) on microgravity-induced IEB injury and explore its underlying mechanism. A rat tail-suspension model was used to simulate microgravity (SMG). Histomorphology, ultrastructure, permeability, and expression of junction proteins in jejunum, ileum, and colon of SMG rats were determined. Proteomic analysis was used to identify differentially expressed proteins (DEPs) in rat ileum mucosa altered by DB. The potential mechanism of DB to protect IEB dysfunction was validated by western blotting. The effects of several components in DB were evaluated in SMG-treated Caco-2 cells. DB protected against IEB disruption by repairing microvilli and crypts, inhibiting inflammatory factors, lowering the permeability and upregulating the expression of tight and adherens junction proteins in the ileum of SMG rats. Proteomic analysis showed that DB regulated 1080 DEPs in rat ileum mucosa. DEPs were significantly annotated in cell–cell adhesion, focal adhesion, and cytoskeleton regulation. DB increased the expression of Rac1-WAVE2-Arp2/3 pathway proteins and F-actin to G-actin ratio, which promoted the formation of focal adhesions. Loureirin C in DB showed a protective effect on epithelial barrier injury in SMG-treated Caco-2 cells. DB could protect against IEB dysfunction induced by SMG, and its mechanism is associated with the formation of focal adhesions mediated by the Rac1-WAVE2-Arp2/3 pathway, which benefits intestinal epithelial cell migration and barrier repair.

## 1. Introduction

The space environment mainly includes microgravity, strong radiation, and high noise, which are hostile factors to the human body. Microgravity (MG) could cause adverse influences on astronauts’ health and working performance and even put their life at risk [1,2,3]. It has been widely reported that MG leads to various kinds of pathophysiological changes, including cardiovascular dysfunction, muscular atrophy, bone loss, and nerve, and immune system disturbance [4,5,6]. Several countries search for effective drugs to protect against pathophysiological injuries induced by MG [7,8,9]. Since multi-components, unique action mechanisms and less toxicity of traditional Chinese medicine (TCM), many kinds of medicinal herbs and TCM combinations have been used in China’s aerospace medicine to treat microgravity-related diseases [10]. Recent several studies show that MG or simulated microgravity (SMG) could change the pathological state of the digestive system. Spaceflight significantly decreased mucin production of intestinal epithelial cells (IECs) in rats [11]. SMG damaged intestinal homeostasis via increasing intestinal permeability [12,13], impairing barrier function and increasing the susceptibility to colitis and the risk of intestinal infection [14,15,16]. This evidence suggests that MG or SMG damages intestinal epithelial barrier (IEB) function. The small intestine is an important organ in the human body. Besides performing the functions of absorption, transportation, metabolism, and immunity, the intestine mucosal structure forms an essential barrier between the external environment and internal milieu, restricting the passage of harmful substances, infectious agents, and microorganisms into circulation in human body [17]. IEB dysfunction could lead to malnutrition, diarrhea, and inflammatory bowel diseases (IBDs) [18], which may increase the intestinal infection risk of astronauts during long-term space travel. To date, potentially therapeutic herbal drugs to protect against IEB dysfunction under microgravity have not been reported.

Dragon’s Blood (DB), a kind of TCM, is a red resin from Dracaena cochinchinensis (Lour.) SC Chen. As an ethnomedicine in China, DB is widely used to treat blood stasis syndrome [19], trauma [20], and ischaemic stroke [21] for several years. The biological activity of DB mainly comes from flavonoids and phenolic constituents, such as loureirin A, B, C, 7,4’-dihydroxyflavone, resveratrol and pterostilbene [19]. Available reports have proved that DB shows therapeutic effects in diabetes [22], acute myocardial infarction [23], stomach ulcer and diarrhoea [24]. DB also exhibits anti-inflammatory, immune inhibitory, anti-cancer and anti-oxidative properties [19,25,26]. In recent years, DB has been used to treat ulcer colitis (UC) in clinical settings [27]. A study proved that DB alleviated dextran sulphate sodium (DSS)-induced acute UC via the RSK/TSC2/mTOR/ribosome pathway in mice colon [28]. Xu et al. (2014) reported the underlying mechanism of action of DB tablets in colitis with systems biology-based approach [29]. However, the mechanism of action of DB components in inhibiting colitis or treating intestinal inflammation remains to be clearly elucidated. In recent years, DB has shown great potential to protect from a physiological injury induced by space radiation or SMG. DB exhibited radioprotective effects in radiation-induced rats and myelosuppressive mice [30]. DB improved blood rheology, inhibited blood coagulation, and prevented oxidative damage of myocardium in SMG rats [31]. However, to the best of our best knowledge, whether DB protects against IEB dysfunction induced by MG remains unexplored. Active constituents in DB or action mechanisms of DB in repairing microgravity-related IEB damage remain unknown.

Owing to the high cost, low frequency and technical difficulties of space experiments, several well-accepted SMG models have been employed for ground-based experiments, including the rat tail-suspension [32], restricted feeding of rabbit head-down [33], human head-down bed-rest [34,35], and rotary cell culture system or random positioning machine for cell culture [36]. Among these, the rat tail-suspension model (also known as the Morey–Holton model) is a validated ground-based spaceflight analog that mimics multiple effects of real microgravity on organisms [14,15] and it has been approved by the National Aeronautics and Space Administration [16].

This study aimed to investigate the effects of DB and its potential therapeutic compounds on IEB damage induced by SMG and explore its underlying molecular mechanism. The rat tail-suspension model and rotary Caco-2 cell culture system were used to simulate microgravity. Histomorphology, ultrastructure, intestinal permeability, inflammatory factors and the expression of tight and adherens junction proteins were determined to evaluate the protective effects of DB. A label-free proteomic strategy was employed to analyze overall changes in rat intestinal mucosa proteins after DB treatment. The potential signaling pathway of IEB dysfunction induced by SMG was validated by western blotting. The effects of potentially active DB compounds on IEB damage were further validated with Caco-2 cells. Finally, the therapeutic effects of DB and its constituents on IEB dysfunction induced by SMG and its underlying mechanism would be first elucidated. This study may prove the novel therapeutic value of DB and provide a novel insight into the mechanism of DB against IEB damage under microgravity conditions. These findings might help prevent the intestinal homeostasis of astronauts during space travel.

## 2. Results

### 2.1. DB Attenuated IEB Injury in SMG Rats

**Figure 1:**
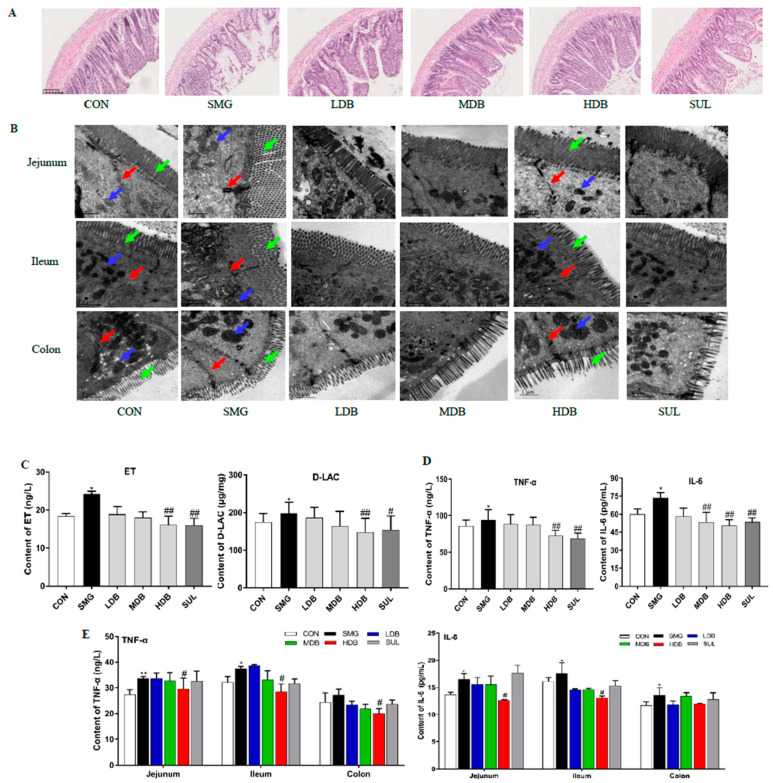
Dragon’s Blood (DB) attenuated intestinal epithelial barrier injury induced by simulated microgravity group (SMG) in rat intestine. (**A**) HE staining of rat ileum tissues in different groups (ruler scale of 100 µm). (**B**) ultrastructure of rat jejunum, ileum, and colon tissue as observed by TEM (green, red and blue arrows represent microvilli, intercellular space and mitochondrial, ruler scale of 1 µm). (**C**) permeability of rat intestinal barrier reflected by D-LAC and ET (*n* = 9). (**D**) the protein levels of TNF-α and IL-6, in rat plasma as determined by ELISA (*n* = 9). (**E**) the protein levels of TNF-α and IL-6 in rat jejunum, ileum, and colon tissues as determined by ELISA (*n* = 9). Note: * *p* < 0.05 and ** *p* < 0.01, compared with CON group. ^#^ *p* < 0.05 and ^##^ *p* < 0.01, compared with SMG group.

To evaluate the DB effect on the histomorphology of different intestinal segments, the jejunum, ileum, and colon of rats were stained with hematoxylin-eosin (HE). Compared with the control group (CON), the histological examination showed that SMG destructed intestinal microvilli and induced erosion and crypt loss in rat ileum (Figure 1A) and jejunum (Supplementary Figure S1A). Submucosal swelling and inflammatory infiltration were observed in the rat ileum, jejunum, and colon (Supplementary Figure S1B) of the SMG group. The ileum and jejunum mucosa showed necrosis and signs of atrophy under SMG conditions. No obvious damage was observed in the colon except inflammatory cell infiltration. These results demonstrated that the ileum was the most severely damaged segment, followed by the jejunum and colon. For DB treatment groups, medium-dose DB (MDB,1 g/kg), high-dose DB (HDB, 2 g/kg) and sulfasalazine (SUL, 0.3 g/kg) significantly alleviated the swelling of the intestinal mucosa, increased the number of crypts and restored the shape of intestinal microvilli in the rat ileum and jejunum. This indicated that MDB, HDB and SUL remarkably reduced the pathological changes in the rat ileum and jejunum and effectively protected the epithelial barrier of ileum and jejunum in SMG rats. HDB inhibited inflammatory infiltration in the colon of SMG rats.

Transmission electron microscopy (TEM) observation revealed that microvilli (green arrow) were broken, and the intercellular space (red arrow) was enlarged in three intestinal segments of the SMG rat ileum and jejunum (Figure 1B). The morphology of mitochondria was incomplete with a damaged membrane in the SMG rat ileum and jejunum (blue arrow, Figure 1B). After the HDB treatment, there was no obvious gap between epithelial cells in three intestinal segments of SMG rats. Intact microvilli, clear cristae, and complete membrane structure of mitochondria were found in the HDB and SUL groups (Figure 1B). These indicated that HDB could decrease the permeability of intestinal epithelium and improve the damaged ultrastructure in the SMG rat intestine. DB significantly alleviated the IEB injury induced by SMG.

### 2.2. DB Decreased Permeability and Inflammation in SMG Rat Intestine

Endotoxin (ET) and D-Lactate have been used as indices to reflect the high permeability of intestinal epithelia [37]. As shown in Figure 1C, 28d-SMG remarkably increased plasma D-LAC and ET concentrations (*p* < 0.05, compared with the CON group). After SMG rats were orally administrated with three doses of DB, DB significantly decreased the levels of D-LAC and ET in the rat plasma in a dose-dependent manner (*p* < 0.01, compared with the SMG group). This result indicated that HDB could reduce the high permeability of the small intestine under SMG conditions. SMG could also trigger the inflammatory responses in rats, and the elevated inflammatory factors can aggravate the intestinal barrier dysfunction [38]. Figure 1D showed that HDB remarkably decreased TNF-α tumor necrosis factor-α (TNF-α) and interleukin-6 (IL-6) plasma levels in SMG rats (*p* < 0.01, compared with the SMG group). Increased TNF-α levels in SMG rats’ ileum, jejunum, and colon were reduced by HDB. HDB decreased the IL-6 content in SMG rat’s ileum and jejunum (Figure 1E). These results indicated that DB decreased permeability and attenuated inflammation in SMG rats. DB at 2 g/kg exhibited the best protective effect on IEB dysfunction induced by SMG and contributed most to the rat ileum and jejunum repair, followed by the rat colon.

### 2.3. DB Protected Intestinal Barrier by Increasing the Expression of TJ and AJ Proteins in SMG Rats

Tight junction (TJ)and adherens junction (AJ) play very important roles in maintaining the integrity of the intestinal barrier [39]. In this study, the expressions of three TJ proteins (zona occluden-1, occludin, claudin-1) and two AJ proteins (endothelial-cadherin and β-catenin) were determined by western blotting. Since the internal controls, such as glyceraldehyde-3-phosphate dehydrogenase (GAPDH), α-tubulin, and β-actin, were not stable in this study, the relative expression levels of proteins were expressed as the ratio of the grey value of the target band to that of total proteins in the same sample [40]. The gels of the total protein of samples are shown in Supplementary Figure S2.

**Figure 2:**
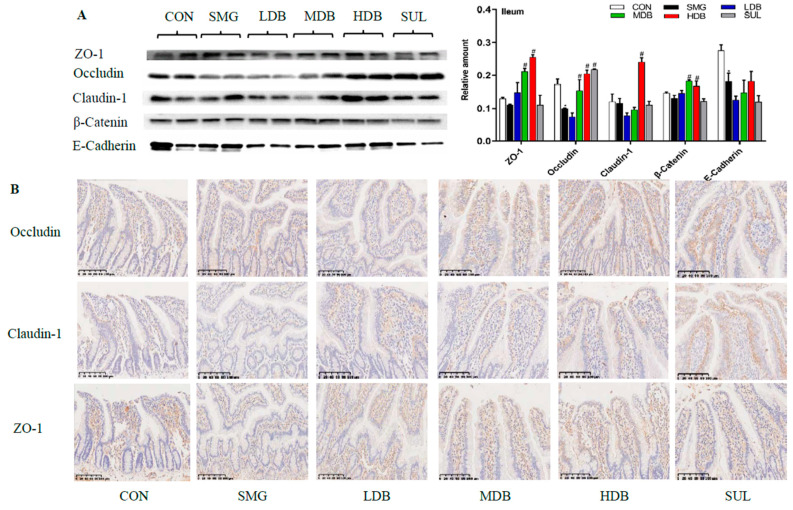
DB protected the intestine epithelial barrier (IEB) injury via increasing the expression of TJ and AJ proteins in ileum of SMG rat. (**A**) the expression of ZO-1, occludin, claudin-1, β-catenin and E-cadherin determined by Western-blot (*n* = 9). (**B**) the expression of occludin, claudin-1and ZO-1 detected by immunohistochemistry (brown granules represent TJ proteins) in SMG rat ileum tissues (ruler scale of 100 µm, *n* = 9). Note: * *p* < 0.05, compared with CON group. ^#^ *p* < 0.05, compared with SMG group. The relative expression levels of proteins were expressed as the ratio of gray value of the target band to that of total proteins in the same sample. The total protein gel was shown in Supplementary Figure S2.

Western blotting showed that occludin, endothelial-cadherin (E-cadherin) and β-catenin levels in jejunum and ileum mucosa of SMG rats were dramatically decreased (*p* < 0.05) compared with those in the CON group. Zona occluden-1 (ZO-1) and claudin-1 did not show significant change in the ileum mucosa of SMG rats. HDB treatment dramatically upregulated the expressions of ZO-1, occludin, claudin-1, and β-catenin in the SMG rat jejunum mucosa compared with those in the SMG group (*p* < 0.05, Figure 2A). Consistently, the immunohistochemistry (IHC) analysis indicated that SMG induced a significant decrease in the expressions of ZO-1 and occludin. IHC results also demonstrated that HDB promoted the expressions of ZO-1 and occludin in the ileum of SMG rats (Figure 2B). Expressions of TJ and AJ proteins in the rat jejunum and colon determined by western blotting and IHC are shown in Supplementary Figure S3. HDB only increased E-cadherin expression in the rat jejunum (Supplementary Figure S3A) and significantly promoted the expression of AJ and TJ proteins, except ZO-1, in the rat colon (Supplementary Figure S3B). IHC results showed a conclusion similar to the western blotting results (Supplementary Figure S3C,D). All the above results indicated that DB protected IEB injury by promoting the expressions of TJ and AJ proteins, which is critical to maintain the optimal intestine integrity under SMG conditions. In summary, according to the results of histomorphology, ultrastructure, inflammation cytokines, and expressions of TJ and AJ proteins, it has been observed that the ileum is the most sensitive intestinal segment to SMG and HDB showed the better protective effect on the SMG-induced injury than the jejunum and colon. Thus, the rat ileum tissue with a high dose of DB administration was selected for further proteomic analysis.

### 2.4. DB Significantly Altered Intestinal Mucosa Proteins in SMG Rats Based on Proteomics

#### 2.4.1. Identification of Differentially Expressed Protein (DEPs)

**Figure 3:**
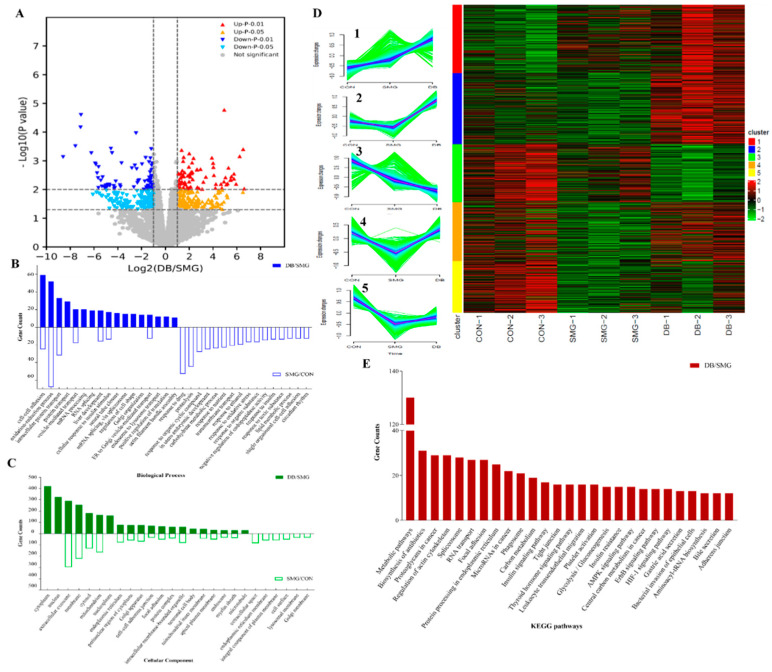
Differentially expressed proteins (DEPs) in ileum of SMG rats with DB treatment were clustered and functionally annotated. (**A**) the volcanic map of DEPs in DB/SMG group. (**B**) biological process classification of DEPs. (**C**) cellular component classification of DEPs. (**D**) heatmap of DEPs with 5 clusters. (**E**) KEGG pathways of DEPs in DB/SMG group.

To investigate the changed proteins and potential pathways of DB on IEB dysfunction induced by 28 days of SMG, a label-free proteomic strategy was applied to detect DEPs in rat ileum samples from SMG and DB (2 g/kg) groups. A total of 5757 proteins were identified, and those identified in DB and SMG comparison groups are shown in a volcano plot (Figure 3A). DEPs with fold changes of >1.5 were recognised as upregulation and <0.67 as downregulation with a *p* value of <0.05. Compared with the CON group, 902 DEPs were screened in the SMG group (marked as SMG/CON comparison group). Compared with the SMG group, 1080 DEPs were found in the DB group (marked as DB/SMG comparison group). Compared with SMG groups, 795 upregulated and 50 downregulated proteins were found in the ileum mucosa of rats orally administered with DB. The expression of P-glycoprotein (P-gp) in the SMG group determined by western blotting demonstrated a pattern consistent with the MS analysis (shown in Supplementary Figure S4).

#### 2.4.2. Gene-Ontology (GO) and the Kyoto Encyclopedia of Genes and Genomes (KEGG) Analysis

The proteomic data was further analyzed by the bioinformatics tool DAVID (Version 6.8). The functions of DEPs were annotated according to GO and KEGG pathway analyses. The GO terms of biological process (BP) and cellular component (CC) of all DEPs between SMG/CON and DB/SMG comparison groups were annotated according to gene counts. The first 30% of the annotation are listed in Figure 3B,C. For the SMG/CON comparison group, the BP annotations were mainly assigned to oxidation–reduction process, response to drug, proteolysis, intracellular protein transport, response to organic cyclic compound, cell–cell adhesion and in utero embryonic development. Cytoplasm, nucleus, extracellular exosome, membrane, mitochondrion, focal adhesion, and cell–cell AJ were included in the CC annotation. For the DB/SMG comparison group, the BP annotation showed that the cell–cell adhesion, oxidation–reduction process and intercellular protein transport were the top three significantly enriched biological processes. Moreover, regulation of cell shape, actin filament bundle assembly and cell migration were also found in the BP annotation. CC annotation was assigned to cellular components related to mitochondrion, such as cell–cell AJ and focal adhesion. GO annotation analysis demonstrated that DB significantly regulated cell–cell adhesion, oxidation–reduction and cytoskeleton compared with the SMG group.

To visualize and explore the protein expression patterns in different samples, all DEPs were clustered using the Mfuzz package (www.bioconductor.org, 24 December 2020). Five clusters were generated (Figure 3D). Compared with the CON group, DEPs in Cluster 2, 4, and 5 were downregulated in the SMG group, which indicated that SMG could lead to an abnormal change in these proteins. DB treatment could potentially upregulate the expression of these proteins back to the normal level. Similarly, DB showed the ability to downregulate some of the increased DEPs in Cluster 1 and 3 in the SMG group. Proteins in Cluster 2, 4, and 5 were further analyzed with the GO term annotation, and it was found that DEPs in Cluster 2 and 4 were mainly involved in biological processes of cell–cell adhesion, AJ, focal adhesion, and mRNA splicing via spliceosome. Cluster 5 was mainly related to metabolic processes, such as lipid homeostasis and fatty acid beta-oxidation.

KEGG pathway enrichment analysis of DEPs in the DB/SMG comparison group showed that metabolic pathways, biosynthesis of antibiotics, proteoglycans in cancer, regulation of actin cytoskeleton, and spliceosome were the top five significantly enriched pathways (Figure 3E). In addition, focal adhesion, AJs, TJs and bacterial invasion of epithelial cell pathways were also observed. Cell adhesions to neighboring cells and surrounding extracellular matrices are essential for various cellular functions, including morphogenesis, migration, proliferation, and differentiation [41]. The actin cytoskeleton is directly connected to TJs and AJs and plays an important role in the assembly and maintenance of these junction structures [42]. Focal adhesions (FAs) and multi-protein complexes act as transmembrane links between extracellular matrices (ECM) and the actin cytoskeleton [43]. Bacterial invasion of epithelial cells is also associated with the regulation of the actin cytoskeleton. Cell adhesion, regulation of the cytoskeleton, and their associated cell migration may play critical roles in maintaining the integrity of intestinal epithelium [44]. Based on the above GO annotation and KEGG pathway results, subsequent analysis mainly focused on the DEPs related to cell adhesion, regulation of the cytoskeleton, focal adhesion, TJs and AJs and bacterial invasion of epithelial cell pathways in the DB/SMG comparison group.

#### 2.4.3. DB Regulated Proteins Related to IEB Damage in the Intestinal Mucosa of SMG Rats

All the DEPs related to cell adhesion, regulation of cytoskeleton, focal adhesion, TJs and AJs and bacterial invasion of epithelial cell pathways in the DB/SMG comparison group are listed in Table 1.

Cell adhesion represents the cell–cell (such as AJ and TJ) and the extracellular-matrix adhesions. Disrupted TJs and AJs have been commonly found in intestinal diseases, including inflammation and malabsorption [45]. Proteomic data indicated that expressions of ZO-1, ZO-3, claudin 2 and 3, and α-catenin in the DB group were upregulated. Furthermore, western blotting showed that DB could increase expressions of ZO-1, occludin, β-catenin, and claudin-1 in the ileum of SMG rats. AJ and TJ could connect the cytoskeletons of neighboring cells. Cytoskeleton directly or indirectly links both cell–ECM and cell–cell adhesion. The cell–ECM adhesion is essential to maintain cell morphology or cell migration [46]. Integrins play critical roles in the linkage between the actin cytoskeleton and ECM. Integrins could connect with actin filaments through linker or adaptor proteins, including α-actinin, filamin, paxillin, and zyxin. Several integrin subunits could regulate intestinal epithelial function under pathological conditions [47]. The expressions of β1 and β4 integrins were downregulated with reduced clustering of FAs in malignant human MCF-7 cells subjected to SMG [48]. In this study, DB increased the expression of integrin α6, β4, α-actinin, filamin, and zyxin in SMG rats, indicating that DB may promote an actin–integrin–ECM linkage and contribute to maintaining intestinal epithelial cell morphology or migration.

Our results also showed that FAs in SMG rats were significantly regulated by DB treatment. FAs could mediate the interaction of cells with ECM. FAs are useful for cell migration, differentiation, and repair of the IEB. Key roles of the FA formation include new nucleation, site-directed branching and elongation of actin filaments. Actin-related protein 2/3 (Arp2/3) is one major determinant nucleator protein to nucleate branched actin filaments [49]. In this study, it has been found that the Arp2/3 subunit 5 was significantly decreased in the SMG group. Several proteins involved in the regulation of Arp2/3 nucleation that significantly changed by the DB treatment include talin, filamin B, zyxin, and diaphanous-related formin 1 (Diaph1). These work as linker proteins that regulate FA formation. Nucleation of actin filaments is useful to produce lamellipodium, which could facilitate membrane protrusion and adhesion at the leading cell edge during cell migration [50]. Our proteomic analysis also indicated that lamellipodium and filopodium were dramatically enriched in cellular components in the DB/SMG comparison group. HE results showed that intestinal crypts were damaged under SMG, which indicates that intestinal stem cells could not migrate to the intestinal cavity or could not differentiate into different intestinal mucosal cells. Consequently, ageing IECs cannot be renewed in time and the IEB would be damaged. Based on the above analysis, it is speculated that DB regulates FA formation through the Arp2/3-mediated nucleation of the branched actin filament, which benefits intestinal epithelial cell migration.

### 2.5. DB Protected IEB Dysfunction in SMG Rats via the Rac1-WAVE2-Arp2/3 Signaling Pathway

Dynamic changes in actin polymerization can regulate FAs. Actin polymerization refers to the aggregation of globular-actin (G-actin, actin monomer) into filamentous-actin (F-actin). The F-actin to G-actin ratio explains the dynamic changes in actin polymerization [51]. Nucleation by Arp2/3 is prominently regulated by the Wiskott–Aldrich syndrome family of nucleation promoting factors, such as the WASP family verprolin-homologous protein 2 (WAVE2). WAVE2 could bind Arp2/3 complex to generate branched filaments to form FAs [52]. The Ras-related C3 botulinum toxin substrate 1 (Rac1) could recruit and activate WAVE2. Blocking the Rac1-WAVE2-Arp2/3 signaling pathway in tumor cells would suppress cell migration [53]. Crk/dedicator of cytokinesis 180 (Dock180) can activate Rac1 and regulate cell migration [54]. To further validate whether DB protects against IEB injury via the Rac1-WAVE2-Arp2/3 signaling pathway, expression of Dock180, Rac1, WAVE2 and Arp3 were determined by western blotting. The F-actin to G-actin ratio was also determined to evaluate the actin polymerization/depolymerization state.

**Figure 4:**
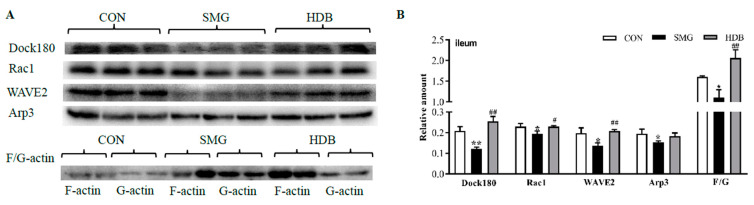
DB increased the expression of Dock180, Rac1, Arp3, WAVE2, and the ratio of F-actin to G-actin in rat ileum. (**A**) the Western-blot bands of Dock180, Rac1, WAVE2, Arp3, and F-actin and G-actin. (**B**) semi-quantitative analysis based on the relative density of the validated proteins. Data was normalized with total protein amount as the internal reference (*n* = 9). It indicated DB treatment could dramatically increase expression of these proteins. Note: * *p* < 0.05 and ** *p* < 0.01, compared with CON group. ^#^ *p* < 0.05 and ^##^ *p* < 0.01, compared with SMG group. The relative expression levels of proteins were expressed as the ratio of gray value of the target band to that of total proteins in the same sample. The total protein gel was shown in Supplementary Figure S5.

Compared with the CON group, SMG treatment showed that Dock180, Rac1, WAVE2 and Arp3 decreased by 41.3%, 14.4%, 31.1%, and 21.0% (*p* < 0.05), respectively (shown in Figure 4). DB treatment could dramatically upregulate the expression of these proteins with the fold of 51.9% (Dock180), 14.4% (Rac1), 34.4% (WAVE2), and 15.6% (Arp3) (*p* < 0.05). The F-actin to G-actin ratio in the DB group increased by 46.6% compared with that in the SMG group. These results demonstrated that DB could promote FA formation through regulating the Rac1-WAVE2-Arp3 pathway, which may contribute to the migration of IECs and help repair epithelial barrier damage under SMG conditions.

### 2.6. Effect of LC, LA, LB, and 7,4’-DHF on SMG-Induced IEB Injury in Caco-2 Cells

As the potentially active compounds in DB, LC, LA, LB, and 7,4’-DHF were used to evaluate the protective effect on SMG-induced IEB injury in Caco-2 cells. Downregulated expression of TJ, AJ and Rac1-WAVE2-Arp3 pathway proteins and damaged ultrastructure were used to confirm that the 48 h-SMG exposure could cause significant barrier injury in Caco-2 cells (Supplementary Figure S6). CCK-8 assay showed that 40 μM of LA and LB, 35 μM of LC and 55 μM of 7,4’-DHF did not affect cell viability. Three doses of LC could significantly upregulate the expression of ZO-1, occludin and claudin-1 in SMG-treated Caco-2 cells. LC at 10 μM increased the expression of E-cadherin and β-catenin. Three doses of 7,4’-DHF could significantly upregulate the expression of β-catenin and claudin-1 in Caco-2 cells subjected to 48 h-SMG. Moreover, 7,4’-DHF at 2.5 μM increased the expression of E-cadherin and β-catenin. 7,4’-DHF showed the potential to upregulate the expression of ZO-1 but without significance. LA (2.5 and 5.0 μM) showed the ability to upregulate the expression of occludin and E-cadherin in Caco-2 cells (without significance). Three doses of LB did not improve the expression of AJ or TJ proteins in Caco-2 cells (data was not shown). Therefore, LB was not selected for further pathway validation experiments. These results are presented in Supplementary Figure S7.

**Figure 5:**
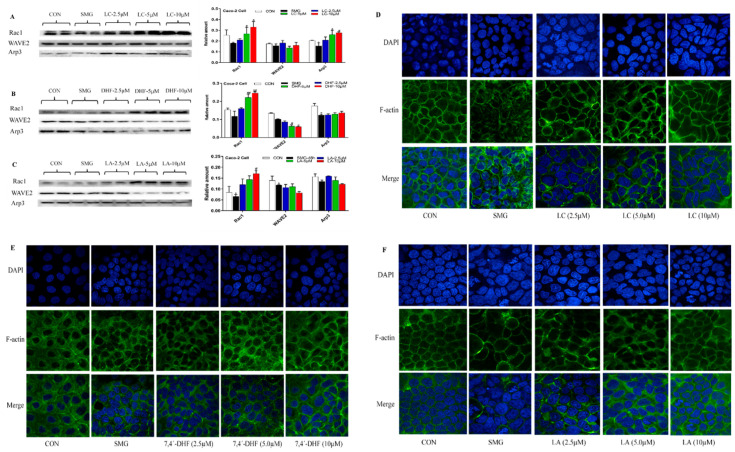
LC (**A**),7,4’-DHF (**B**) and LA (**C**) regulated the expression of Rac1-WAVE2-Arp3 pathway proteins in SMG-treated Caco-2 cells. Effect of LC (**D**),7,4’-DHF (**E**) and LA (**F**) on rearrangement of F-actin. Note: * *p* < 0.05, compared with CON group. ^#^ *p* < 0.05 and ^##^ *p* < 0.01, compared with SMG group. The relative expression levels of proteins were expressed as the ratio of gray value of the target band to that of total proteins in the same sample. The total protein gel was shown in Supplementary Figure S7.

LC treatments at 5 and 10 μM significantly upregulated the expression of Rac1 and Arp3 in a dose-dependent manner, whereas LC did not dramatically affect the expression of WAVE2 (Figure 5A). A high dose of LA and 7,4’-DHF dramatically increased the expression of Rac1 and did not promote the expression of Arp3 (Figure 5A,B). The expression of Dock180 and the F-actin to G-actin ratio were also determined after SMG-treated Caco-2 cells were incubated with LC, LA and 7,4’-DHF. However, three compounds did not remarkably change the expression of Dock180 and the F-actin to G-actin ratio. Immunofluorescence analysis showed that LC (2.5, 5, and 10 μM) and LA (5 and 10 μM) could improve the irregular distribution of F-actin and increase the number of actin filaments in SMG-treated Caco-2 cells (Figure 5D–F). Three doses of 7,4’-DHF did not show remarkable influence on the distribution of F-actin (Figure 5E). Collectively, LC and 7,4’-DHF may show the potential to upregulate the expression of TJ and AJ proteins in Caco-2 cells subjected to SMG. LC exhibits a better regulatory effect on the Rac1-WAVE2-Arp3 pathway and distribution of F-actin in SMG-treated Caco-2 cells than 7,4’-DHF and LA.

**Figure 6:**
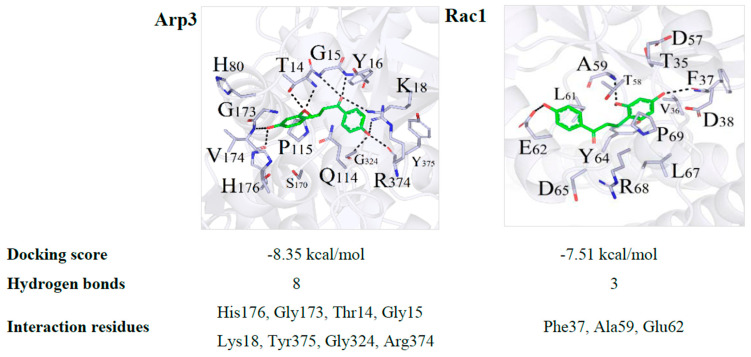
Interaction modes of LC with the binding pocket of Rac1 and Arp3. The black-dashed lines indicate hydrogen bonds with the residues in the binding cavity of proteins.

To further explore the interaction of LC with Rac1, WAVE2, and Arp3, molecular docking between LC and Rac1, WAVE2, and Arp3 were performed following the described method using the AutoDock 4.2 software [55]. Since there was no integrity crystal structure for WAVE2, the molecular docking between LC and WAVE2 was not conducted. Three-dimensional binding conformations of the LC with Rac1 and Arp3 with the lowest docking energy are presented in Figure 6. The docking energy scores were −8.35 and −7.51 kcal/mol for Rac1 and Arp3, respectively. Dynamics simulation showed that LC was embedded into the canonical ligand-binding cavity of Rac1 and Arp3. LC formed eight hydrogen bonds with the amino acid residues His176, Gly173, Thr14, Gly15, Lys18, Tyr375, Gly324, and Arg374 in Arp3. LC interacted with Rac1 at the amino acid residues Phe37, Ala59, and Glu62 (Figure 6). LC showed the binding activity with Arp3 and Rac1. These amino acid residues might be potentially active sites of LC.

## 3. Discussion

It has been observed that medium and high doses of DB (1 and 2 g/kg) have protective effects on the histomorphology and ultrastructure of the ileum and jejunum in SMG rats. Although no serious damage was observed in the histomorphology of the SMG rat colon, DB (2 g/kg) could significantly improve the ultrastructure and increase the expression of TJ and AJ proteins in the SMG rat colon (Supplementary Figure S3B). However, DB could not effectively increase the expression of ZO-1, occluding, and β-catenin in the jejunum of SMG rats (Supplementary Figure S3B). These results may imply that DB exhibits different effects in the three intestinal segments of SMG rats. The underlying reasons need further study. Based on the results of histomorphology, ultrastructure, levels of inflammatory factors, and expressions of TJ and AJ proteins, the present study selected the ileum mucosa of SMG rats administered with 2 g/kg of DB for proteomic study.

Our previous study has reported that the IEB damage in the rat jejunum barrier under 21 days of SMG conditions might be associated with increased myosin light chain kinase (MLCK) phosphorylation level [15]. However, we have confirmed that DB could not protect the IEB damage in rat jejunum, ileum and colon via the MLCK phosphorylation related pathway (such as RhoA/Rock/MLCK pathway). Based on the pharmacoproteomic analysis, it has been found that DB remarkably regulates cell adhesion, especially FA formation. FA formation is regulated by pathways associated with the Arp2/3 complex [52]. The linkage of the actin cytoskeleton to ECM at FAs provides a physical path for cells to exert traction forces on substrates during cell migration and morphogenesis [49]. This is achieved by the local accumulation of actin filaments and linker proteins (such as zyxin, talin, and vinculin) that connect actins to integrins. Inhibited Rac1-WAVE2-Arp2/3 signaling pathway could reduce the migration of U251 human glioma cells and MCF10A epithelial cells with a significantly reduced focal adhesion assembly [50,56]. Microgravity could reduce cell adhesion and FA formation, which affects cell migration capacity and viability via downregulated expression of actin and actin-associated proteins, such as Arp2/3 [57].

Results of proteomic analysis indicate that DB could dramatically regulate linker proteins, including zyxin, filamin, talin, and vinclin (listed in Table 1). DB increases the F-actin to G-actin ratio and the expression of Rac1-WAVE2-Arp2/3 pathway proteins (Figure 4), which suggests that DB promotes FA formation by regulating actin polymerization and increasing the accumulation of actin filaments at FAs. This would benefit intestinal epithelial cell migration. Although the rat colon epithelial barrier injury induced by SMG is not quite serious, it is still noteworthy that DB could effectively protect rat colon damage through the Rac1-WAVE2-Arp2/3 pathway (data not shown). Supplementary Figure S1 shows that DB could repair the damaged jejunum barrier induced by SMG. However, it has been confirmed that DB exhibits protective effect not via regulating the Rac1-WAVE2-Arp2/3 pathway in rat jejunum. This may imply that DB shows distinct mechanisms of action in the three intestinal segments of SMG rats. The underlying reason needs further investigation.

To screen potentially active compounds in DB and identify DB components in plasm and the ileum of SMG rats orally administered with 2 g/kg of DB, the ultra-liquid chromatography-high resolution-MS (UPLC-HR-MS) method was employed. Six prototype components (LA, LB, LC, 7,4′-DHF, resveratrol and pterostilbene) were identified. Molecular docking analysis of six in vivo DB components and 65 proteins (DEPs, TJ, and AJ proteins and Rac1-WAVE2-Arp2/3 pathway proteins are shown in Table 1) were performed following the described method of the AutoDock 4.2 software [55]. The molecular docking energy scores and chemical structures of six compounds are listed in Supplementary Table S1. There were no crystal structures for actinin alpha 4 (Actn4), diaphanous-related formin 1 (Dia1), solute carrier family 9 member A1 (Slc9a1), and radixin (Rdx). There were no integrity crystal structures for cingulin (Cgn), symplekin (Sympk) and WAVE2. Thus, these seven proteins did not show docking energy score in Supplementary Table S1. According to optimal docking energy scores, it has been found that LC, LA, LB, and 7,4′-DHF are the top four compounds that could bind large numbers of DEPs (Table 1). Dcok180, Rac1, and Arp3 were mainly targeted by LC, LA, LB, and 7,4′-DHF. Consequently, LC, LA, LB, and 7,4′-DHF were selected to validate the protective effect on an SMG-induced IEB damage in Caco-2 cells. Compared with LA, 7,4′-DHF and LB (results in Section 2.6), LC shows the best potential to increase the expression of TJ and AJ proteins and regulate the Rac1-WAVE2-Arp3 pathway in SMG-induced Caco-2 cells. LC could interact with Rac1 or Arp3 via multi-hydrogen bonds and amino acid residue sites (Figure 6). If specific inhibitors of Rac1 or Arp3 or site-directed mutation cell model were used for further investigation, it would be more useful to explore the IEB protection effect of LC.

Pharmacokinetics of LC, LA, LB, and 7,4′-DHF in SMG rats orally administered with DB have been investigated in our group [31]. These compounds showed decreased absorption, slower elimination, and double peaks in concentration–time curves in SMG rats. Metabolism of LB in the SMG rat liver microsome; LA, LC, DHF in the human liver microsome, and altered expression of drug-metabolizing enzymes in liver and intestine of SMG rats have been reported in our previous studies [58,59,60]. Altered drug-metabolizing enzymes and PK behaviors of DB compounds may be related to the therapeutic effects of DB in SMG rats.

## 4. Materials and Methods

### 4.1. Materials

DB (voucher specimen number: 20160701) was supplied by the Ligong-Genyuan Technology Company (Beijing, China) and was identified by Dr. Yulin Deng of School of Life Sciences, Beijing Institute of Technology. The voucher specimen was preserved in the School of Life Science, Beijing Institute of Technology. The levels of loureirin A, B, C, and 7,4′-dihydroxyflavone in DB were 0.43%, 0.40%, 0.38% and 0.61%, respectively [31] (Wang et al., 2021). Loureirin A (LA, CAS:119425-89-7), loureirin B (LB, CAS:119425-90-0), loureirin C (LC, CAS:116384-24-8) and 7,4′-dihydroxyflavone (7,4′-DHF, CAS: 2196-14-7) were obtained from Shanghai Yuanye Biological Technology Company (Shanghai, China, purity > 98%). Sulfasalazine (Lot number: H31020840) was purchased from Shanghai Fuda Pharmaceutical Company (Shanghai, China).

The antibodies of E-cadherin (Cat.#: AB76055, 1:1000 dilution), β-catenin (Cat.#: AB32572, 1:5000), zona occluden-1 (ZO-1, Cat.#: AB190085, 1:1000), claudin-1 (Cat.#: AB15098, 1:1000), occludin (Cat.#: AB216327, 1:5000), Arp3 (Cat.#: AB181164, 1:5000) and Rac1 (Cat.#: AB33186, 1:5000) were obtained from Abcam (Cambridge, MA, USA). The anti-rabbit and mouse IgG (Cat.#: C030205, 1:5000) were purchased from Dakemei Technology Company (Shanghai, China). Dock180 (Cat.#: C4C12, 1:1000) was purchased from Cell Signaling Technology (Danvers, MA, USA). WAVE2 (Cat.#: YT4898, 1:1000) was purchased from ImmunoWay Biotechnology Company (Plano, TX, USA).

### 4.2. Animal Treatment and Sample Collection

Fifty-four SPF male Sprague–Dawlery (SD) rats (body weight: 200 ± 20 g) were purchased from Beijing HFK Bioscience Co. Ltd. All animal studies complied with the Guide for the Care and Use of Laboratory Animals published by the National Institutes of Health (NIH publication No.85-23, revised in 1996). All experiments were approved by the Beijing Institute of Technology Animal Care and Use Committee (SYXK-BIT-20200109002, 9 Janurary 2020). The animals were maintained under a 12 h light/dark cycle at 24 °C ± 2 °C and relative humidity of 55% ± 5%. Rats had free access to water and food.

The animals were acclimatized for 1 week. All rats were then randomly categorized into six groups of nine each as follows: the control group (CON), simulated microgravity group (SMG), DB group with a low-dose of 0.5 g/kg (LDB), a middle-dose of 1 g/kg (MDB) and a high dose of 2 g/kg (HDB), and the sulfasalazine group (SUL, used as positive control) with a dose of 0.3 g/kg. Drug solutions were prepared by dissolving DB and SUL in 0.5% carboxymethylcellulose-Na (CMC-Na) to obtain the desired concentration. The rats were tail-suspended to ensure that their hind limbs are above off the ground and produce a 30° head-down tilt according to the Morey–Holton model, except for the CON group [32]. Rats were orally administered with DB for 28 consecutive days. After 28 d of tail-suspension, all rats were feed-deprived overnight with free access to water. The rats were anaesthetized with 10% chloral hydrate (350 mg/kg), and blood samples were then directly collected from the heart into heparinized tubes centrifuged at 4000 rpm (5 min, 4 °C) to collect the plasma. Rat jejunum, ileum, and colon mucosa were collected and stored at −80 °C for further analysis. Rat jejunum, ileum, and colon segments were immersed in 4%-paraformaldehyde for haematoxylin and eosin (HE) staining and in glutaraldehyde paraformaldehyde for transmission electron microscopy (TEM) observation.

### 4.3. Enzyme-Linked Immune Sorbent Assay (ELISA)

Approximately 0.1 g of rat jejunum, ileum, and colon mucosa were mixed with a lysis buffer solution and then crushed in an ultrasonic cell disruptor for 3 min. After the homogenate was centrifuged at 4000 rpm for 10 min at 4 °C, the resulting supernatant was collected. The protein concentration of each sample was determined by the Bradford method. The concentrations of endotoxin (ET) and D-Lactate (D-LAC) in the homogenate and inflammatory factors, including tumor necrosis-α (TNF-α) and interleukin-6 (IL-6) in plasma and intestine homogenate, were determined using ELISA kits (Hengyuan Biological Technology Company, Shanghai, China) according to the manufacturer’s instructions.

### 4.4. Histomorphology and Ultrastructure Observation

Different rat intestinal segments (jejunum, ileum, and colon) were embedded in paraffin. The paraffin sections were cut into 4-μm-thick pieces and stained with HE. Histological images of the intestinal tissue were scanned using Nanozoomer S210 microscopic-resolution scanner and observed using the NanoZoomer Digital Pathology View 2.0 software (Hamamatsu Photonics, Shizuoka, Japan).

Intestinal segments were cut into slices of 1 cm^3^ and fixed in cool 4% glutaraldehyde for > 4 h. After washing with phosphate buffer saline (PBS), tissues were fixed in 1% osmic acid, followed by dehydration with gradient acetone and embedding in resin. Finally, tissues were cut into ultrathin sections. Sections were stained using uranyl acetate followed by lead citrate. The ultrastructure of intestine segments was recorded under the JEM-1400 transmission electron microscope (JEOL Ltd., Tokyo, Japan).

### 4.5. Western Blot Analysis

The homogenate, as described in Section 2.4, was mixed with 4× loading buffer (Solarbio, Beijing, China) and boiled for 10 min. The total protein was separated by sodium dodecyl sulphate-polyacrylamide gel electrophoresis (SDS-PAGE) and was then transferred onto polyvinylidene difluoride membranes (PVDF, Merck Millipore Ltd., Burlington, MA, USA). The membranes were blocked in 5% skim milk for 2 h and were then incubated with primary antibodies at 4 °C overnight. The membranes were washed in Tris-buffered saline-tween (TBST) and subsequently incubated with HRP-conjugated anti-rabbit/mouse IgG at room temperature for 2 h. After incubation, the membranes were washed in TBST and colour was then rendered using an enhanced chemiluminescence (ECL) reagent (Millipore, Burlington, MA, USA). The Image Lab TM software (version 3.0, Bio-rad, Hercules, CA, USA) was used to collect the grey value of each strip. The relative expression levels of proteins were expressed as the ratio of the grey value of the target band to that of total proteins in the same sample [40].

### 4.6. Immunohistochemistry Analysis

The expressions of occludin, claudin-1, and ZO-1 were detected by immunohistochemistry (IHC) analysis. Briefly, all blocks of rat jejunum, ileum, and colon tissue were sectioned (4 µm), deparaffinised, rehydrated with gradient ethanol, and finally incubated in fresh 3% hydrogen peroxide for 15 min. The sections were repaired under high pressure at 140 °C for 3 min. After rinsing with PBS, the sections were incubated with primary antibodies (occludin, 1:200; claudin-1, 1:400; and ZO-1, 1:400) overnight at 4 °C. Subsequently, the sections were incubated with HRP-conjugated anti-rabbit and goat IgG at 37 °C for 20 min and colour was then rendered by DAB Immunohistochemistry Color Development Kit. IHC images were scanned by the Nanozoomer S210 microscopic-resolution scanner (Section 2.5).

### 4.7. Proteomics

#### 4.7.1. Sample Preparation

The ileum mucosa from each rat was homogenized in a lysis buffer (containing 7M urea, 2M thiourea and 0.1% 3-[(3-Cholamidopropyl)-dimethyl-ammonio]-1-propane sulfonate) using tissue micro-grinders. The homogenate was subjected to ultrasonic fragmentation and then centrifuged at 15,000 g at 4 °C for 10 min to collect the supernatant. The protein concentration of each sample was determined by the Bradford method. Three biological replicates in CON, SMG and DB groups were separately prepared for further analysis.

#### 4.7.2. Protein Extraction and In-Gel Digestion

The protein samples and loading buffer were proportionally mixed and heated at 100 °C for 8 min to completely denature the protein. According to the original protein concentration, each sample was diluted to 1 μg/μL using a lysis buffer. Samples were separated by a 5% stacking gel and 12% separating gel of SDS-PAGE with a sample size of 30 μg. After electrophoresis, the separating gel was placed in Coomassie Blue fast staining solution for 1 h and then decolorized until clear and obvious bands appeared on the gel.

The gel was cut into strips along the lane, and each strip was evenly cut into five pieces into tubes for enzymatic digestion. The enzymatic digestion method was as per a previously described protocol [15]. Briefly, the gel pieces were decolourized using a decolorising solution. Gel pieces were then rehydrated with dithiothreitol (DTT) at 56 °C for 30 min, darkly alkylated with iodoacetamide (IAM) for 30 min and then enzymolysized by trypsin overnight. Trifluoroacetic acid (TFA) was added to terminate the reaction. Finally, a dry peptide powder was obtained by rapid vacuum concentration and stored at −80 °C.

#### 4.7.3. RP-HPLC-MS/MS Analysis

The dry peptide samples were dissolved in a methanol-H_2_O-formic acid (2%: 98%: 0.1%, v:v:v) solution and centrifuged at 13,000 rpm for10 min to collect the supernatant. A peptide sample of 10 μL each was separated using Thermo Scientific EASY-nLC 1200 (Nano-HPLC) chromatographic system coupled to Thermo Scientific Fusion Lumos mass spectrometer. A C18 reverse-phase column (3 µm, 150 mm × 75 µm, Eksigent Technologies, Silicon Valley, CA, USA) was selected as the stationary phase. Mobile phase A was water containing 0.1% formic acid, and mobile phase B was acetonitrile containing 0.1% formic acid. The flow rate of the mobile phase was 350 µL/min. The separation gradient was set as follows: 0–5 min, 4%–15% B; 5–40 min, 15%–25% B; 40–65 min, 25%–35% B; 65–70 min, 35%–95% B; 70–85 min, 95% B; 8–90 min, 95%–4% B.

An EASY-spray source was used for ionising peptides with a 2100 V spray voltage. The declustering potential was 100 V, and the capillary temperature was 250 °C. The resolution, automatic gain control target and maximum injection time (MAX.IT) for full scan MS were 70,000 Full-Width-Half-Maximum (FWHM), 1000000, and 60 ms, respectively, and 17,500 FWHM, 5,000,000 and 70 ms, respectively, for data-dependent MS (2). The scan range of the full scan MS was 350–1800 m/z. The intensity threshold of dd-MS2 was 5000 m/z. High energy collision dissociation was used as the fragmentation method, and normalized collision energy was 29%. The top 20 precursor ions with the highest abundance in each spectrum were selected for data-dependent MS (2).

#### 4.7.4. Protein Identification and Bioinformatic Analysis

The MaxQuant software (version 1.5.2.8, Max Planck Institute of Biochemistry, Munich, Bavaria, Germany) and UniProt Database (https://www.uniprot.org/, 31 August 2020) were used to search the mass data and analyze the protein sequence. The parameters were set as following: species was Rattus norvigicus. Peptides and proteins were identified if the probability of a false discovery rate was <0.01. The fixed modification was carbamidomethyl, and the alterable modification was oxidation. Trypsin was used as the digestive enzyme, and most two cleavages were missed. The Proteome Discoverer software (version 2.4, Thermo Scientific, Walsham, MA, USA) was used as the retrieval software, and the protein abundance was calculated based on the extracted ion chromatogram (XIC) area. The ratio of protein abundance in every two groups was defined as the fold change. When the fold change was >1.5 or <0.67, with a *p* value of <0.05, the protein was identified to be upregulated or downregulated differentially expressed protein (DEPs). All DEPs were then analyzed using the DAVID software (version 6.8, https://david.ncifcrf.gov/, 10 September 2020, Laboratory of Human Retrovirology and Immunoinformatic, Frederick, MD, USA) for Gene-Ontology (GO) and the Kyoto Encyclopaedia of Genes and Genomes (KEGG) pathway classification.

### 4.8. Caco-2 Cell Experiment with Compounds in DB

#### 4.8.1. Cell Culture and Microgravity Simulation

Caco-2 cells were purchased from the Chinese Academy of Sciences (Shanghai, China) and cultured in an MEM/EBSS medium (Cat.#: SH30024.01, Hyclone, Logan, UT, USA) supplemented with 10% foetal bovine serum, 100 U/mL streptomycin, 100 U/mL benzylpenicillin and 1% non-essential amino acids (Gibco, Grand Island, NY, USA) in a humidified, 5% CO_2_ atmosphere incubator at 37 °C. After Caco-2 cells were cultured for 21 days under normal gravity, cells were seeded in a T-25 flask (2 × 10^4^ cells/cm^2^). The T-25 flask was carefully filled with a culture medium, ensuring no bubbles were formed to avoid shearing of fluid. The Caco-2 cells were exposed to SMG using a 3D clinostat (<10^−3^ g) (National Space Science Center, Beijing, China), whereas the control group was treated at 1 g in the same CO_2_ incubator.

#### 4.8.2. Effect of LA, LB, LC, and 7,4′-DHF on SMG-Treated Caco-2 Cells

LA, LB, LC and 7,4′-DHF were dissolved in DMSO (the final concentration was 0.01%) and diluted with a medium to obtain the desired concentrations. The effect of all compounds on cell viability was analyzed with CCK-8 assay (Cell Counting Kit-8, Cat.#: CA1210, Solarbio, Beijing, China). LA, LB, LC, and 7,4′-DHF (three doses of 2.5, 5.0 and 10.0 µM, respectively) were added into Caco-2 cells and exposed to SMG for 48 h. The expression of adherens junction (AJ), tight junction (TJ), and pathway proteins in cell samples were determined by western blotting (Section 2.6). F-actin was determined by immunofluorescence staining analysis to demonstrate rearrangement of the cytoskeleton. The Caco-2 cells were fixed in a 4% paraformaldehyde for 20 min at 37 °C. The non-specific binding sites were blocked with a 10% BSA for 30 min at 37 °C. The cells were incubated with phalloidin (1:200, Yeasen Biotech Company, Shanghai, China) for 2 h at room temperature. The cells were then incubated with 4′,6-diamidino-2-phenylindole (DAPI, Bioword, Nanjing, China) at a concentration of 0.1 μg/mL for 10 min at room temperature. Finally, images were observed using Nikon A1 laser scanning confocal microscope (Nikon Corporation, Tokyo, Japan).

### 4.9. Statistical Analysis

Statistical analysis was performed using SPSS 20.0 software (IBM, New York, NY, USA), and the results were expressed as mean ± SD. The difference between the two groups was determined by a one-way analysis of variance, and *p* value of < 0.05 was considered to indicate statistical significance.

## 5. Conclusions

In conclusion, this study first elucidated that DB protects against IEB dysfunction by repairing microvilli and crypts, inhibiting inflammatory factors, lowering the permeability, and upregulating the expression of tight and AJ proteins in the ileum of SMG rats. The underlying mechanism is that DB could promote the formation of FAs mediated by the Rac1-WAVE2-Arp2/3 pathway and benefit from the intestinal epithelial cell migration and epithelial barrier repair. Loureirin C in DB might be an active compound to maintain IEB integrity under SMG conditions.

## Figures and Tables

**Table 1:** DEPs in different KEGG pathways related to focal adhesion, regulation of cytoskeleton, adherens, and tight junctions, and bacterial invasion in epithelial cell in DB/SMG comparison group.

No.	Protein ID	Gene Name	Ratio of DB/SMG	KEGG Pathways
1	P63259	Actin, gamma 1 (Actg1)	0.1166	CYTO SKEL, FA
2	F1LNG5	Phosphoinositide-3-kinase regulatory subunit 1 (Pik3r1)	2.8114	CYTO SKEL, FA
3	A0A0G2JSK5	Integrin subunit beta 1 (Itgb1)	2.6357	CYTO SKEL, FA
4	Q6T487	Actinin, alpha 1 (Actn1)	3.4880	CYTO SKEL, FA
5	Q99MI8	Actinin alpha 4 (Actn4)	1.8886	CYTO SKEL, FA
6	R9PXU6	Vinculin (Vcl)	2.4851	CYTO SKEL, FA
7	F1LQT3	Rho-associated coiled-coil containing protein kinase 2 (Rock2)	2.3299	CYTO SKEL, FA
8	A0A0G2JWJ0	Protein phosphatase 1, regulatory subunit 12A (Ppp1r12a)	4.0816	CYTO SKEL, FA
9	A0A0G2K470	Integrin alpha 2 (Itga2)	2.2878	CYTO SKEL, FA
10	D3ZN37	Rho-associated coiled-coil containing protein kinase 1 (Rock1)	2.4038	CYTO SKEL, FA
11	D3ZZW1	Dedicator of cyto-kinesis 1 (Dock1)	3.5549	CYTO SKEL, FA
12	Q01986	Mitogen-activated protein kinase kinase 1 (Map2k1)	1.6345	CYTO SKEL, FA
13	Q5U2U2	V-crk avian sarcoma virus CT10 oncogene homolog-like (Crkl)	1.9489	CYTO SKEL, FA
14	B5DF62	p21- (RAC1)-activated kinase 4 (Pak4)	1.9673	CYTO SKEL, FA
15	F1LSD3	Integrin subunit beta 4 (Itgb4)	1.9011	CYTO SKEL, FA
16	F1M775	Diaphanous-related formin 1(Dia1)	2.2056	CYTO SKEL, FA
17	G3V667	Integrin subunit alpha 6 (Itga6)	1.7947	CYTO SKEL, FA
18	P35465	p21- (RAC1)-activated kinase (Pak1)	2.1580	CYTO SKEL, FA
19	Q6AYF4	Integrin subunit beta 6(Itgb6)	1.5949	CYTO SKEL, FA
20	P36506	Mitogen-activated protein kinase kinase 2 (Map2k2)	5.2687	CYTO SKEL
21	A0A0G2K472	Cytoplasmic FMR1 interacting protein 1(Cyfip1)	1.8093	CYTO SKEL
22	F1LW74	IQ motif containing GTPase activating protein 2 (Iqgap2)	2.0964	CYTO SKEL
23	O88370	Phosphatidylinositol-5-phosphate 4-kinase type 2 gamma (Pip4k2c)	1.5063	CYTO SKEL
24	P26431	Solute carrier family 9 member A1 (Slc9a1)	6.2933	CYTO SKEL
25	P31977	Ezrin (Ezr)	2.0838	CYTO SKEL
26	P55161	NCK-associated protein 1 (Nckap1)	1.6742	CYTO SKEL
27	Q5WQV5	Radixin (Rdx)	6.2933	CYTO SKEL
28	G3V7Q7	IQ motif containing GTPase activating protein 1 (Iqgap1)	1.5901	CYTO SKEL, AJ
29	F1M2P8	Protein kinase C, alpha (Prkca)	1.9988	FA
30	D4A8D5	Filamin B (Flnb)	2.0076	FA
31	F1LPI5	Laminin subunit beta 3 (Lamb3)	3.0845	FA
32	D4A7U1	Zyxin (Zyx)	6.4350	FA
33	G3V852	Talin 1 (Tln1)	2.5176	FA
34	A0A0G2JT61	Rrb-b2 receptor tyrosine kinase 2 (Erbb2)	2.3015	FA, AJ
35	A0A0G2JYB2	Claudin 2 (Cldn2)	3.5026	TJ
36	A0A0G2K8M3	Tight junction protein 3 (Tjp3)	1.6807	TJ
37	D4A4X4	Cingulin (Cgn)	1.6955	TJ
38	F1LNF0	Myosin heavy chain 14 (Myh14)	1.9354	TJ
39	F1LSH0	Symplekin (Sympk)	2.5214	TJ
40	F1M7H7	Membrane associated guanylate kinase (Magi1)	9.2851	TJ
41	G3V6P7	Myosin, heavy chain 9, non-muscle-like 1 (Myh9l1)	1.7921	TJ
42	Q3ZB99	Tight junction protein 2 (Tjp2)	1.8268	TJ
43	Q4QQT4	Protein phosphatase 2 scaffold subunit A beta (Ppp2r1b)	1.6361	TJ
44	Q5XI34	Protein phosphatase 2 scaffold subunit A alpha (Ppp2r1a)	1.5793	TJ
45	Q63400	Claudin 3 (Cldn3)	2.4558	TJ
46	A0A0G2K2P5	Tight junction protein 1 (Tjp1)	1.9539	TJ, AJ
47	Q66HL2	Cortactin (Cttn)	3.6887	TJ, BACT INVAS
48	A0A0A0MY48	Dynamin 2 (Dnm2)	1.8772	BACT INVAS
49	A0A0G2K4S6	Engulfment and cell motility 1 (Elmo1)	5.6117	BACT INVAS
50	F1LRS8	CD2-associated protein (Cd2ap)	3.8926	BACT INVAS
51	F1M779	Clathrin heavy chain (Cltc)	2.0538	BACT INVAS
52	Q499U2	Engulfment and cell motility 3 (Elmo3)	1.8629	BACT INVAS
53	Q5U302	Catenin alpha 1 (Ctnna1)	2.0367	BACT INVAS, AJ
54	A0A0G2JXW2	SMAD family member 4	2.4516	AJ
55	D3ZZZ9	Catenin delta 1 (Ctnnd1)	2.1570	AJ
56	D4A5C0	Nectin cell adhesion molecule 3 (Nectin3)	1.5708	AJ
57	G3V8P4	Protein tyrosine phosphatase, receptor type, F (Ptprf)	2.5484	AJ
	TJ, AJ and Rac1/WAVE2/Arp2/3 pathway proteins			
58	Q6RUV5	Ras-related C3 botulinum toxin substrate 1 (Rac1)	1.1689	-
59	A0A0G2K5T9	Wiskott-Aldrich syndrome protein family member 2 (WAVE2)	1.5264	-
60	Q4V7C7	Actin-related protein 3 (Arp3)	1.1846	-
61	Q6P6T5	Occludin (Ocln)	2.4612	-
62	P56745	Claudin-1 (Cldn1)	1.4483	-
63	Q9R0T4	E-Cadherin (E-Cdhn)	2.3361	-
64	Q9WU82	β-catenin (Ctnnb)	2.2410	

Note: Regulation of cytoskeleton, focal adhesion, tight junction, adherens junction, bacterial invasions of epithelial cell were abbreviated as CYTO SKEL, FA, TJ, AJ, and BACT INVAS, respectively.

## Data Availability

Not applicable.

## Supplementary Materials

The following are available online at https://www.mdpi.com/1422-0067/22/5/2722/s1. Figure S1: HE figures of rat jejunum and colon tissue (ruler scale of 100 µm). DB could repair jejunum barrier dysfunction by improging microvilli, inhibiting crypt loss, edema and inflammatory cell infiltration (**A**, *n* = 9). Slight inflammatory cell infiltration was observed in colon of SMG rats and DB could improve this injury in colon tissue (**B**, *n* = 9), Figure S2: Western-blot bands of TJ and AJ proteins in rat ileum, colon and jejunum, and their corresponding total protein gels, Figure S3: DB protected the intestinal epithelial barrier injury by increasing the expression of TJ and AJ proteins in the jejunum (**A**) and colon (**B**) tissues of SMG rat (*n* = 9). The expression of occludin, claudin-land ZO-1 detected by immunohistochemistry ( brown granules represent TJ protein) in SMG rat jejunum (**C**) and colon (**D**) tissues (ruler scale of 100 µm ) (*n* = 9). * *p* < 0.05 and ** *p* < 0.01, compared with CON group. ^#^ *p* < 0.05 and ^##^ *p* < 0.01, compared with SMG group. Their corresponding total protein gels in rat colon and jejumum were shown in Supplementary Figure S2, Figure S4: The expression of P-glycoprotein (P-gp) by Western-blot method to demonstrate a pattern consistent with MS analysis, Figure S5: Western-blot bands of Dock180, Racl, WAVE2, Arp3 and its corresponding total protein gels in rat ileum, Figure S6: Expression of TJ and AJ proteins in Caco-2 cells with different duration of microgravity simulation (**A**). Several proteins were down-regulated under 48 h-SMG. TEM images of Caco-2 cells with different duration of microgravity simulation (**B**). The results showed that 48 h-SMG led to most serious damage with broken microvilli and enlarged cellular space. Expression of Racl-WAVE 2-Arp3 pathway proteins in Caco-2 cells with different duration of microgravity simulation (**C**). All proteins except Dock180 were down-regulated under 48 h-SMG. Note: * *p* < 0.05 and ** *p* < 0.01, compared with CON group. The relative expression levels of proteins were expressed as the ratio of gray value of the target band to that of total proteins in the same sample. The total proteins of AJ, TJ, Racl-WAVE2-Arp3 pathway proteins in Caco-2 cells with different duration of microgravity simulation (**D-E**), Figure S7: Expression of AJ and TJ proteins in SMG-treated Caco-2 cells incubated with LC (**A**), 7,4’-DHF (**B**) and LA (**C**). LC and LA increased AJ and TJ proteins better than 7,4’-DHF. The total proteins gel corresponding to every band was listed in **D** (LC), **E** (7,4’-DHF), and **F** (LA), respectively. The Western-blot bands of Racl-WAVE2-Arp3 pathway proteins in SMG-treated Caco-2 and their corresponding total proteins gel of LC (**G**), 7,4’-DHF (**H**) and LA (**I**), Table S1: Docking energy (Kcal-mol) of different differentially expressed proteins and chemical structures of loureirin A, loureirin B, loureirin C, 7,4’-DHF, pterostilbene and resveratrol.

## Abbreviations

DB	Dagon’s Blood
IEB	intestinal epithelial barrier
LA	loureirin A
LC	loureirin C
TEM	transmission electron microscopy
D-LAC	D-lactic acid
IL-6	interleukin-6
E-cadherin	endothelial-cadherin
AJ	adherens junction
DEP	differentially expressed protein
TCM	traditional Chinese medicine
SMG	simulated microgravity
LB	loureirin B
7,4′-DHF	7,4′-dihydroxyflavone
ET	endotoxin
TNF-α	tumor necrosis factor-α
ZO-1	zona occluden-1
TJ	Tight junction
IHC	immunohistochemistry
FA	Focal adhesion
