# Supplementary material for: Dragon’s Blood Regulates Rac1-WAVE2-Arp2/3 Signaling Pathway to Protect Rat Intestinal Epithelial Barrier Dysfunction Induced by Simulated Microgravity
Source: Int J Mol Sci. 2021 Mar 8;22(5):2722. doi: 10.3390/ijms22052722 (PMC7962842; doi:10.3390/ijms22052722)
Supplement: Supplementary file 1 [file ijms-22-02722-s001.pdf]

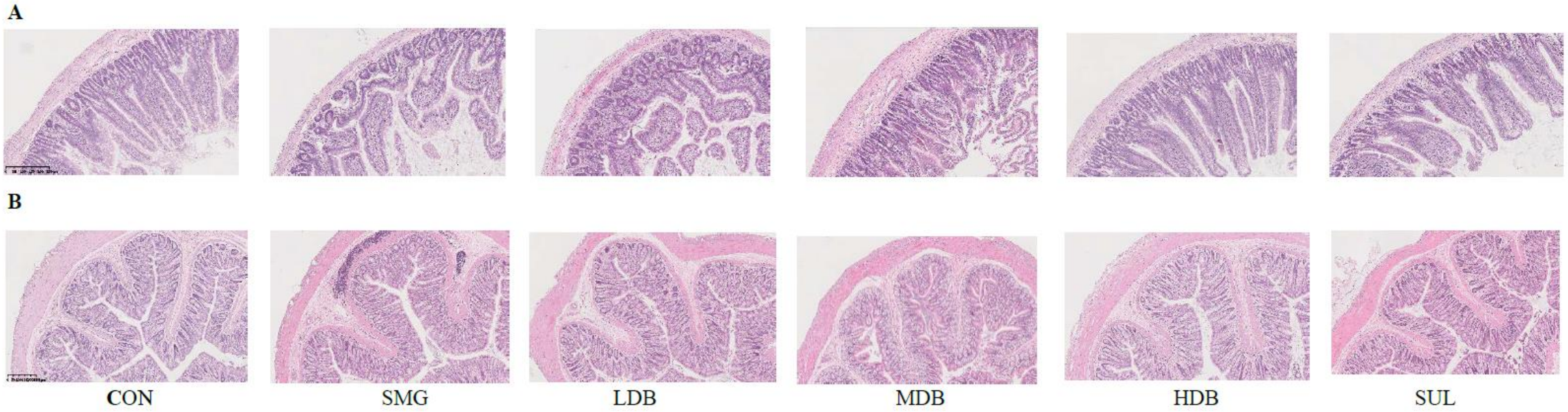

Figure S1. HE figures of rat jejunum and colon tissue (ruler scale of 100 μm). DB could repair jejunum barrier dysfunction by improging microvilli, inhibiting crypt loss, edema and inflammatory cell infiltration (**A**, *n* = 9). Slight inflammatory cell infiltration was observed in colon of SMG rats and DB could improve this injury in colon tissue (**B**, *n* = 9).

Ileum

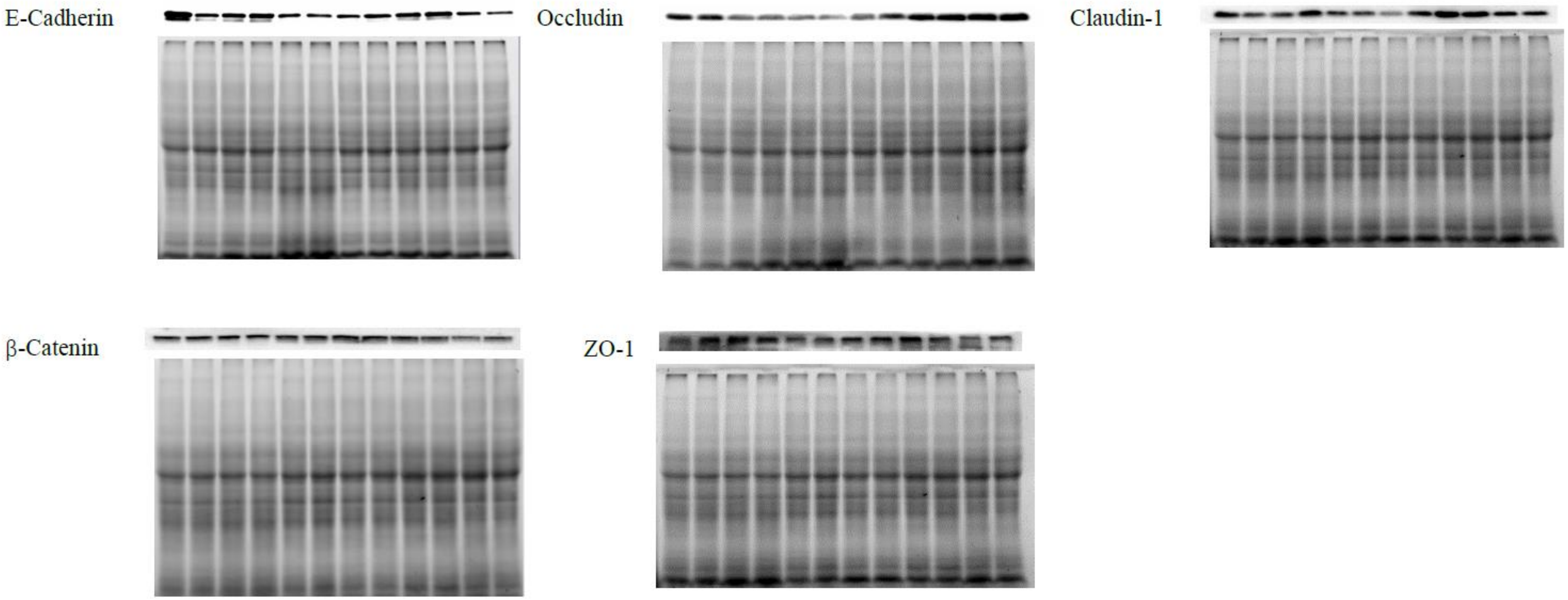

Colon

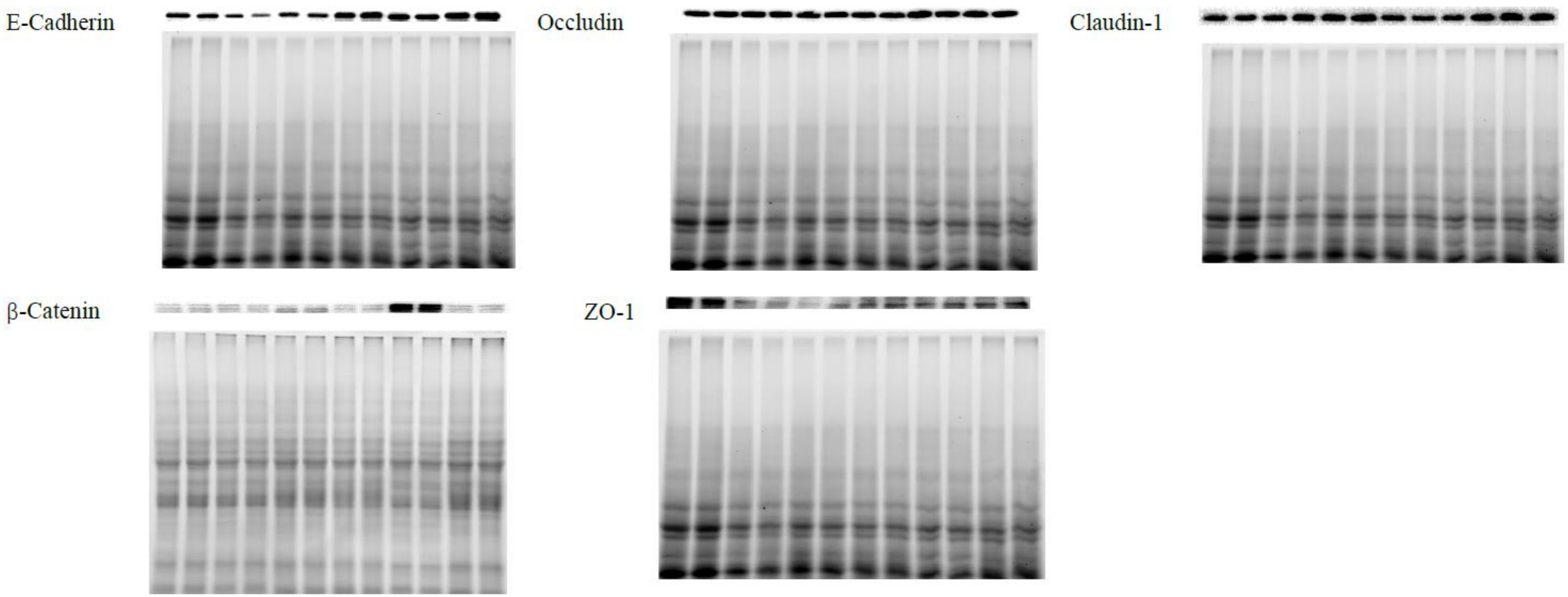

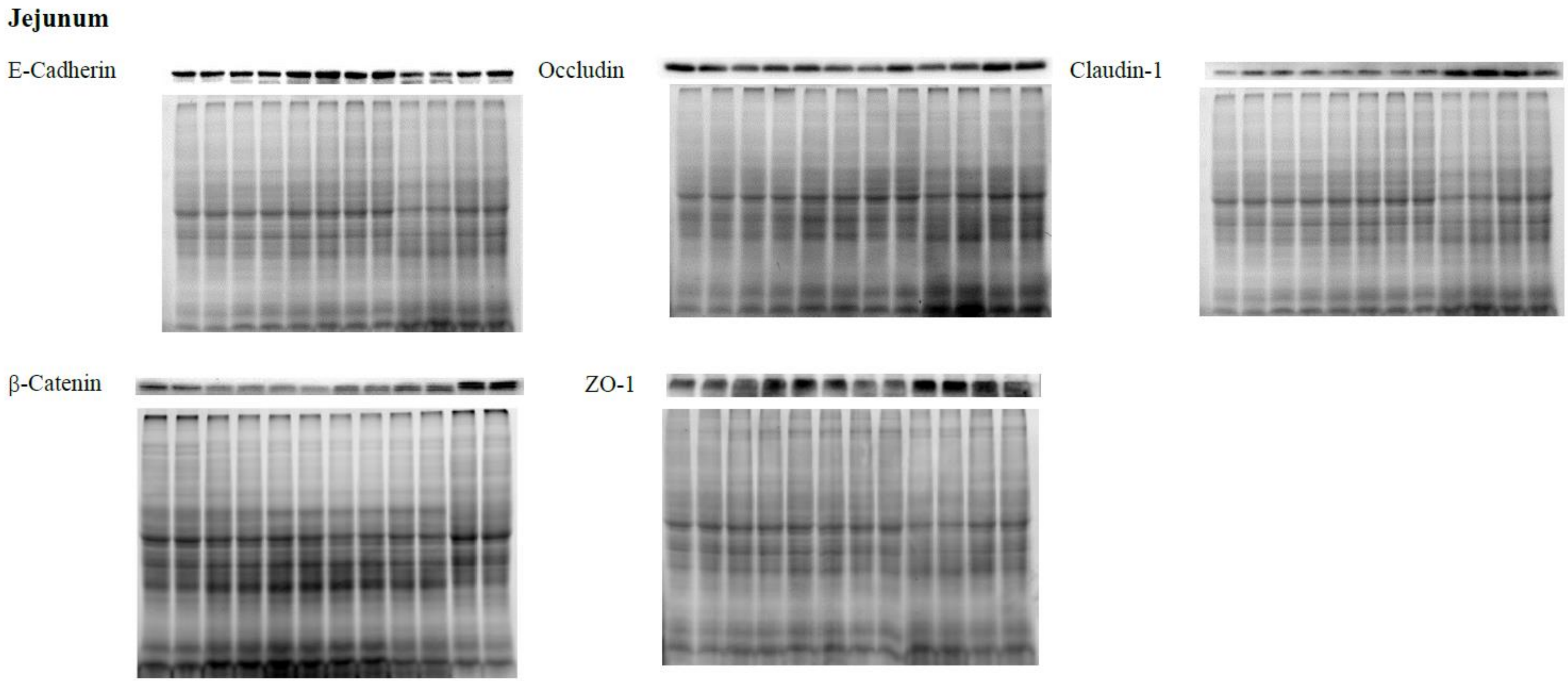

Figure S2. Western-blot bands of TJ and AJ proteins in rat ileum, colon and jejunum, and their corresponding total protein gels.

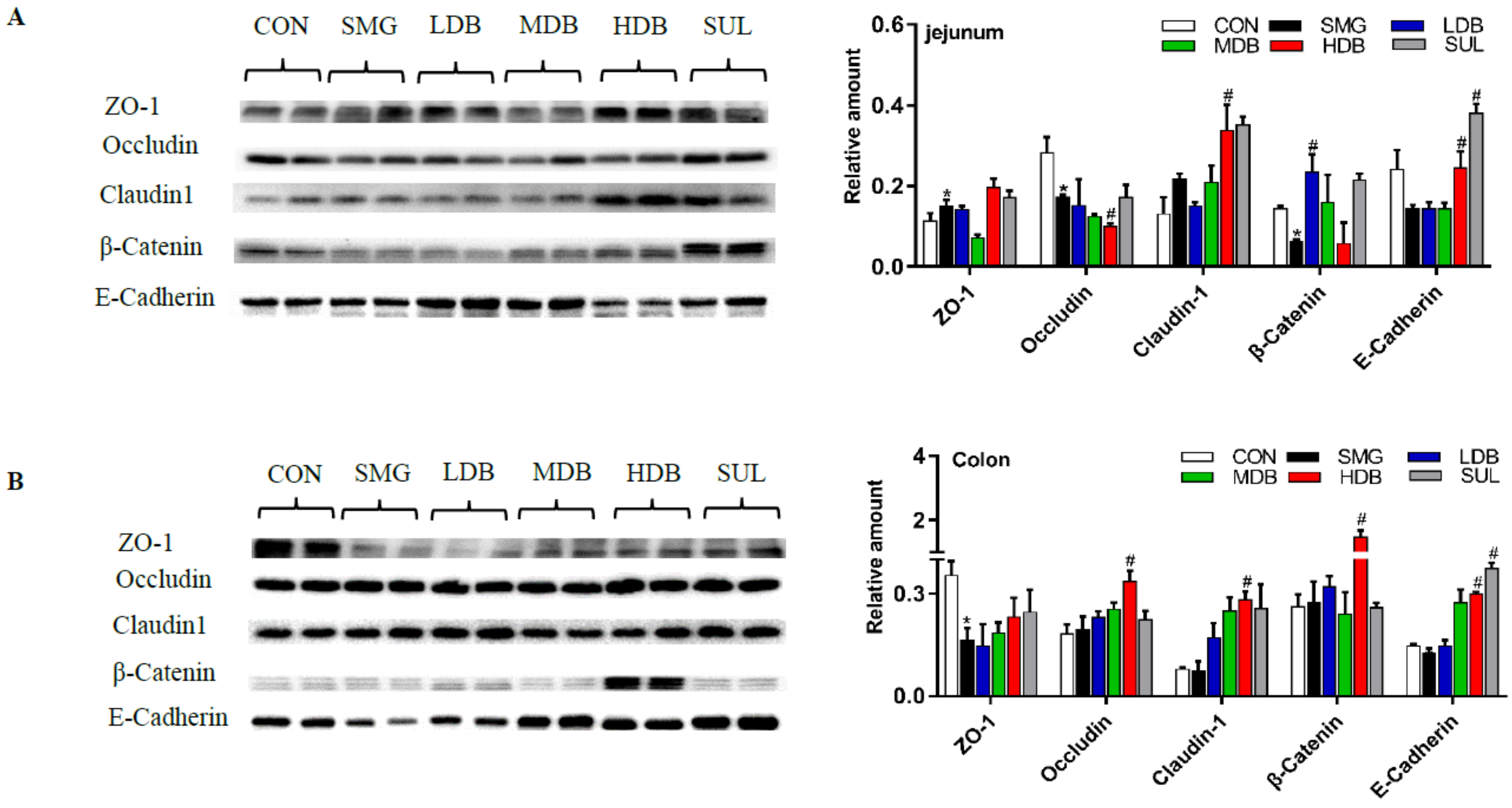
A
CON SMG LDB MDB HDB SUL
ZO-1
Occludin
Claudin1
β-Catenin
E-Cadherin
jejunum
CON SMG LDB MDB HDB SUL
Relative amount
0.6
0.4
0.2
0.0
ZO-1
Occludin
Claudin-1
β-Catenin
E-Cadherin
B
CON SMG LDB MDB HDB SUL
ZO-1
Occludin
Claudin1
β-Catenin
E-Cadherin
Colon
CON SMG LDB MDB HDB SUL
Relative amount
4
2
0.3
0.0
ZO-1
Occludin
Claudin-1
β-Catenin
E-Cadherin

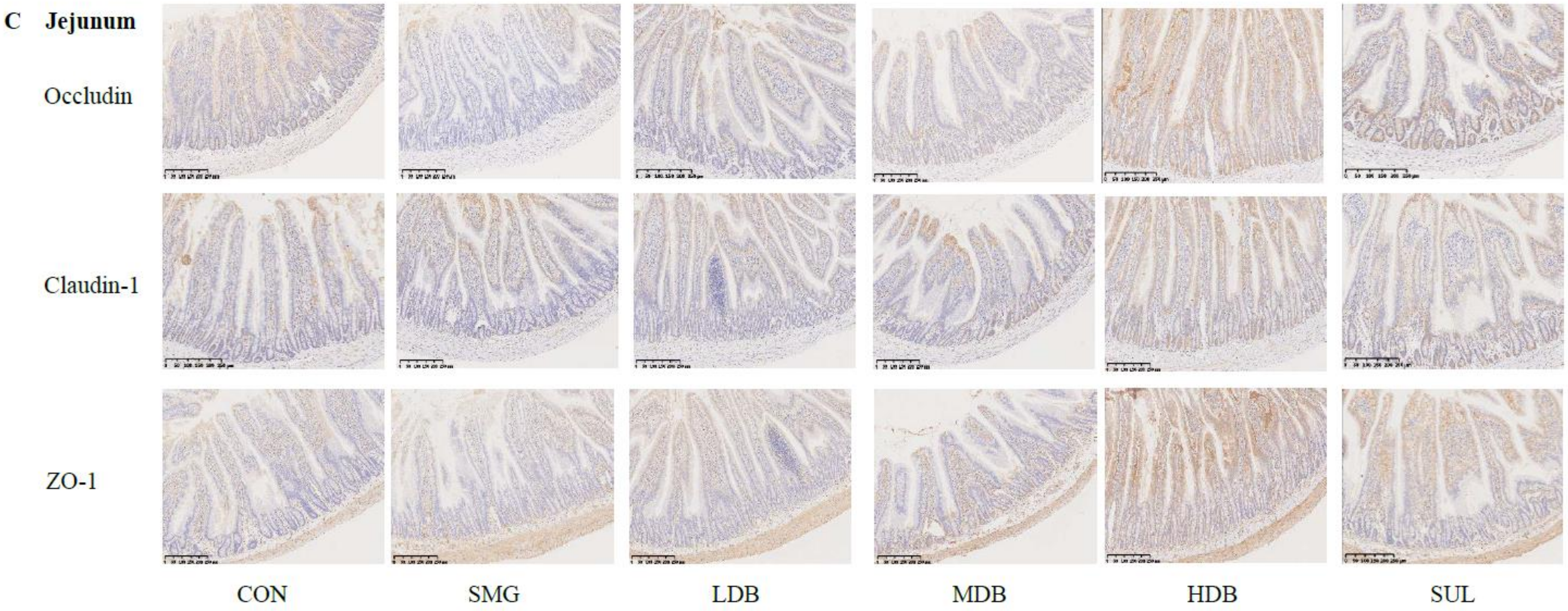
C
Jejunum
Occludin
Claudin-1
ZO-1
CON
SMG
LDB
MDB
HDB
SUL

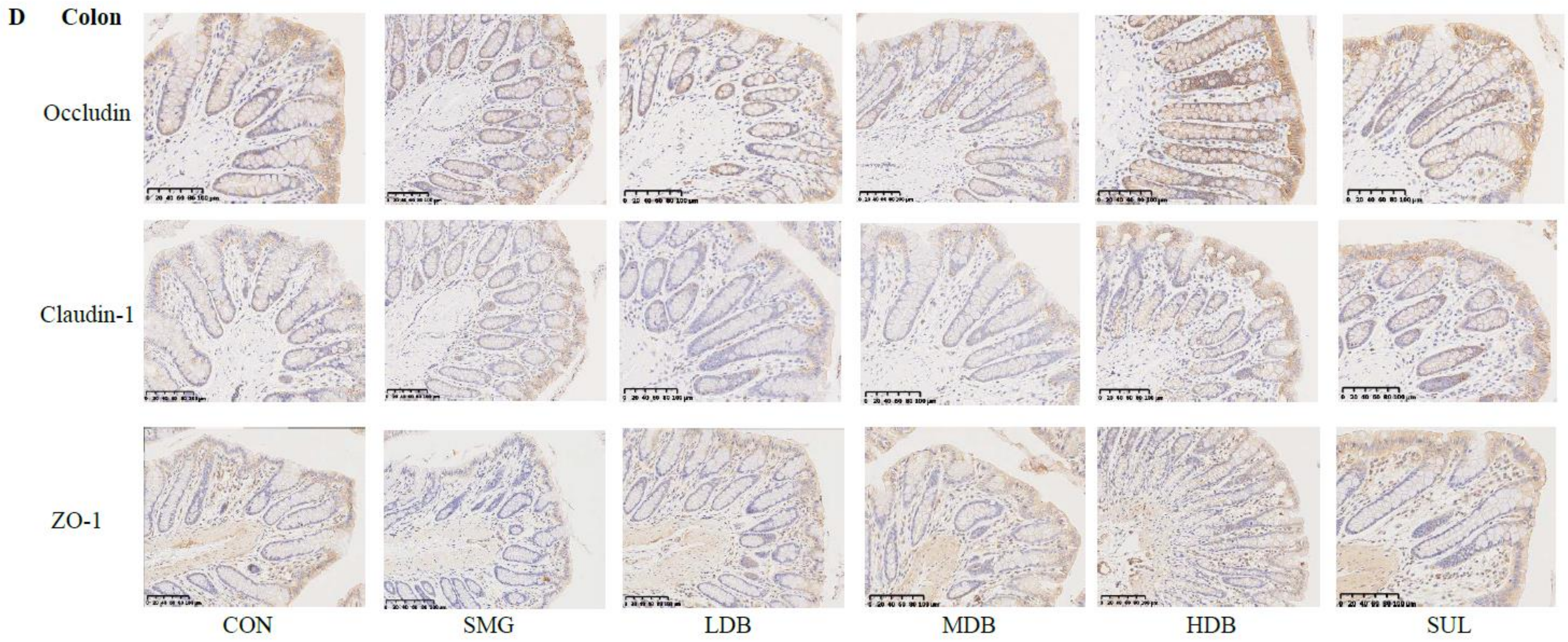

Figure S3. DB protected the intestinal epithelial barrier injury by increasing the expression of TJ and AJ proteins in the jejunum (**A**) and colon (**B**) tissues of SMG rat (n = 9).The expression of occludin ,claudin-land ZO-1 detected by immunohistochemistry (brown granules represent TJ protein) in SMG rat jejunum (**C**) and colon (**D**) tissues (ruler scale of 100 μm) (n = 9). * $p < 0.05$ and ** $p < 0.01$, compared with CON group. # $p < 0.05$ and ## $p < 0.01$, compared with SMG group. Their corresponding total protein gels in rat colon and jejumum were shown in Supplementary Figure S2.

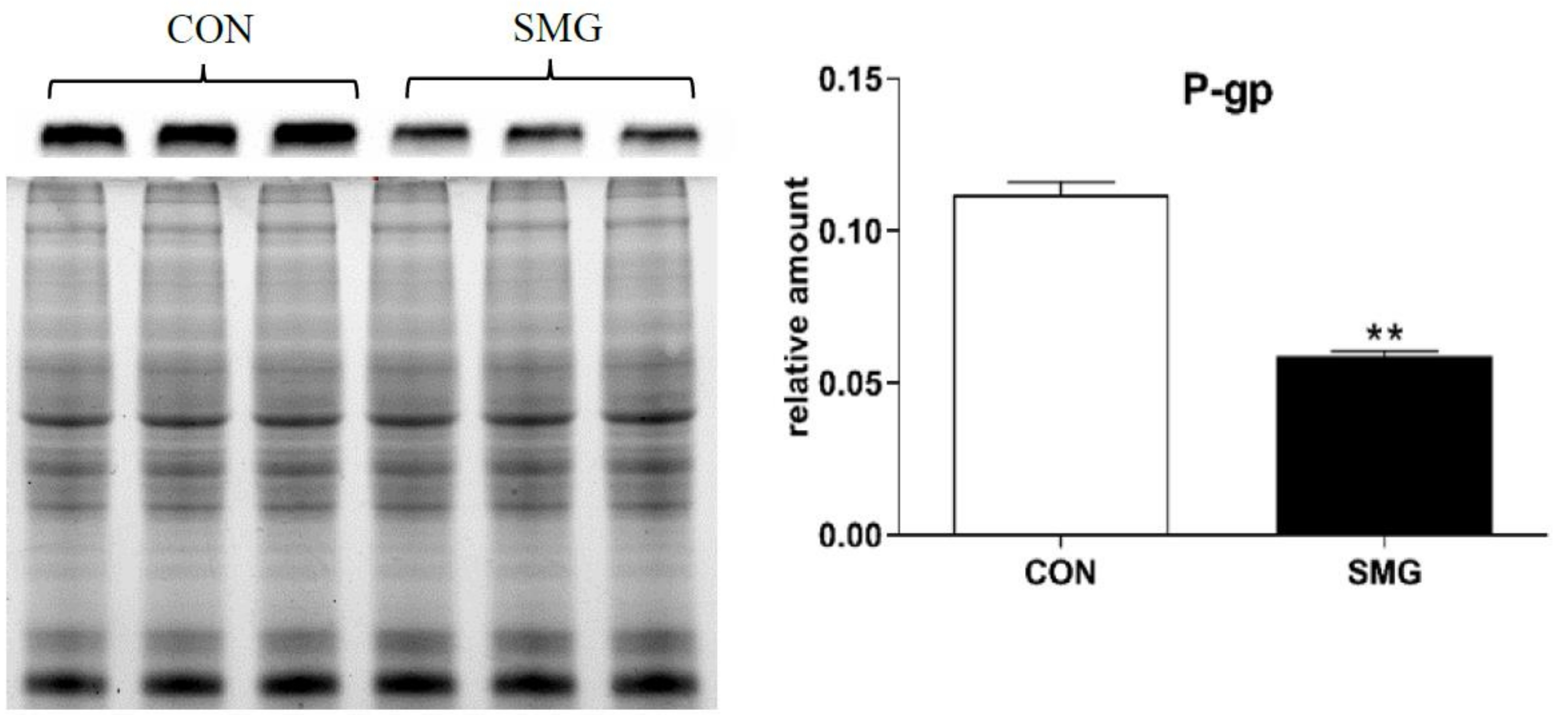

Figure S4. The expression of P-glycoprotein (P-gp) by Western-blot method to demonstrate a pattern consistent with MS analysis.

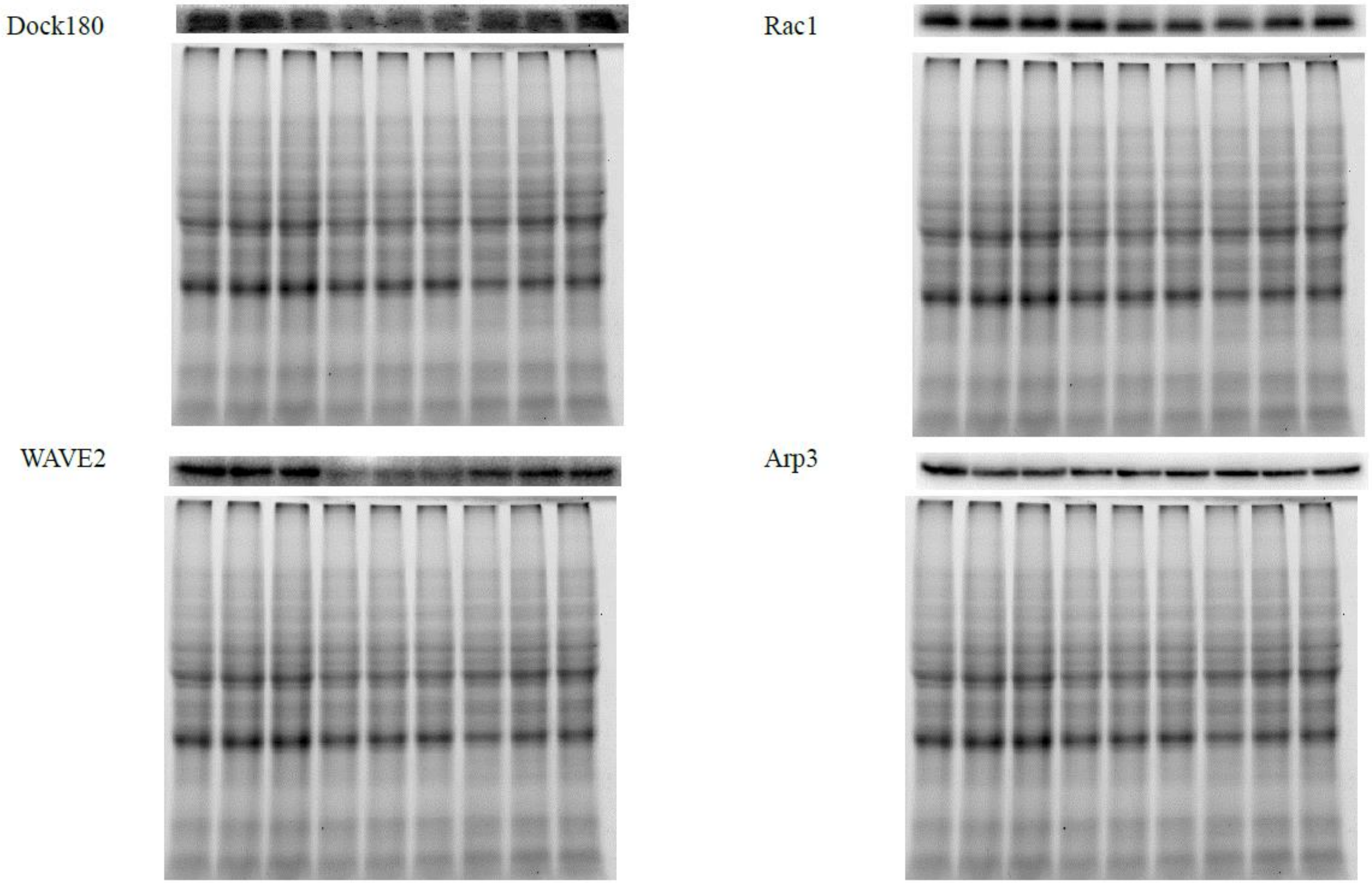

Figure S5. Western-blot bands of Dock180, Racl, WAVE2, Arp3 and its corresponding total protein gels in rat ileum.

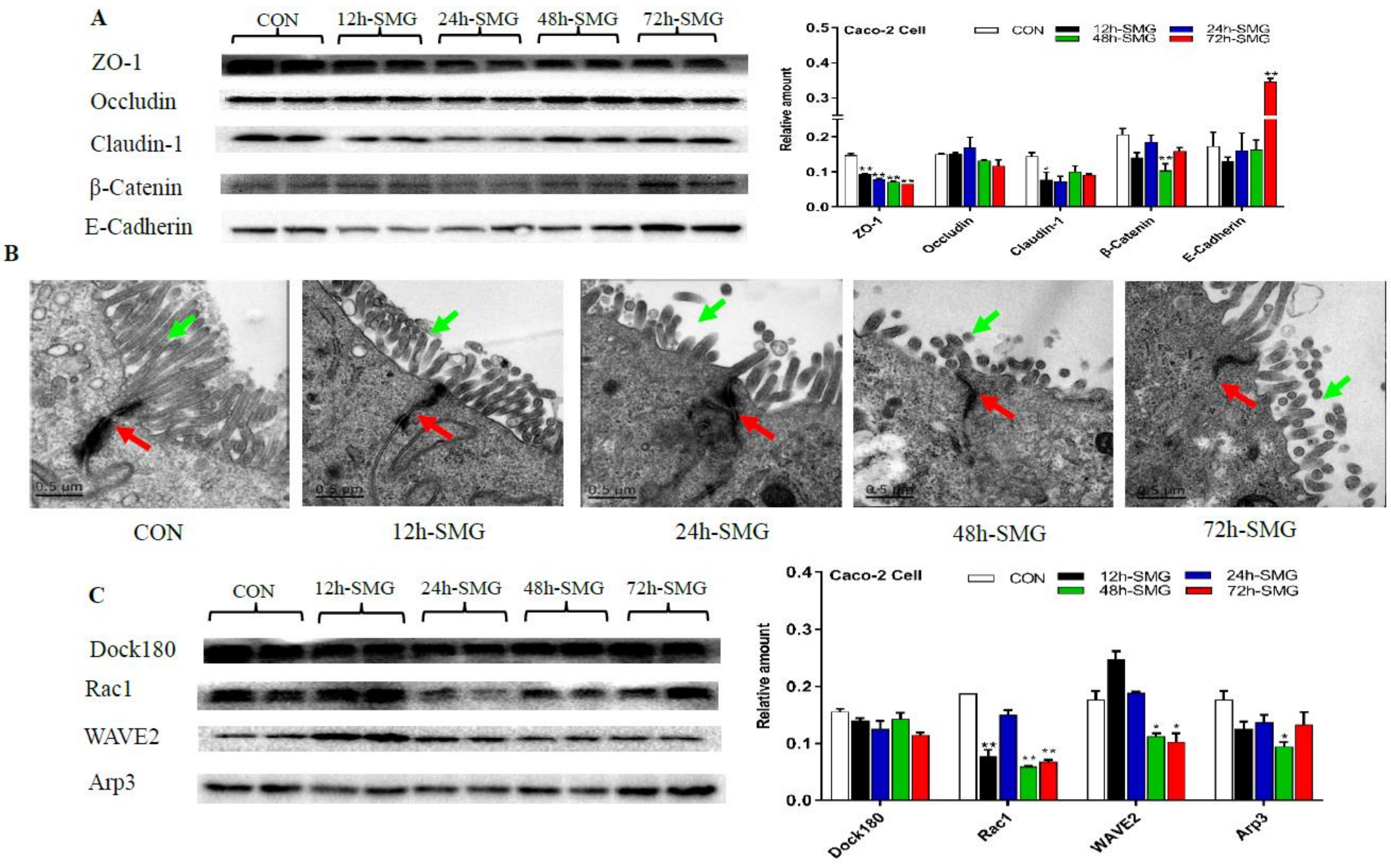

A
CON
12h-SMG
24h-SMG
48h-SMG
72h-SMG
ZO-1
Occludin
Claudin-1
β-Catenin
E-Cadherin
Caco-2 Cell
Relative amount
B
CON
12h-SMG
24h-SMG
48h-SMG
72h-SMG
C
Dock180
Rac1
WAVE2
Arp3

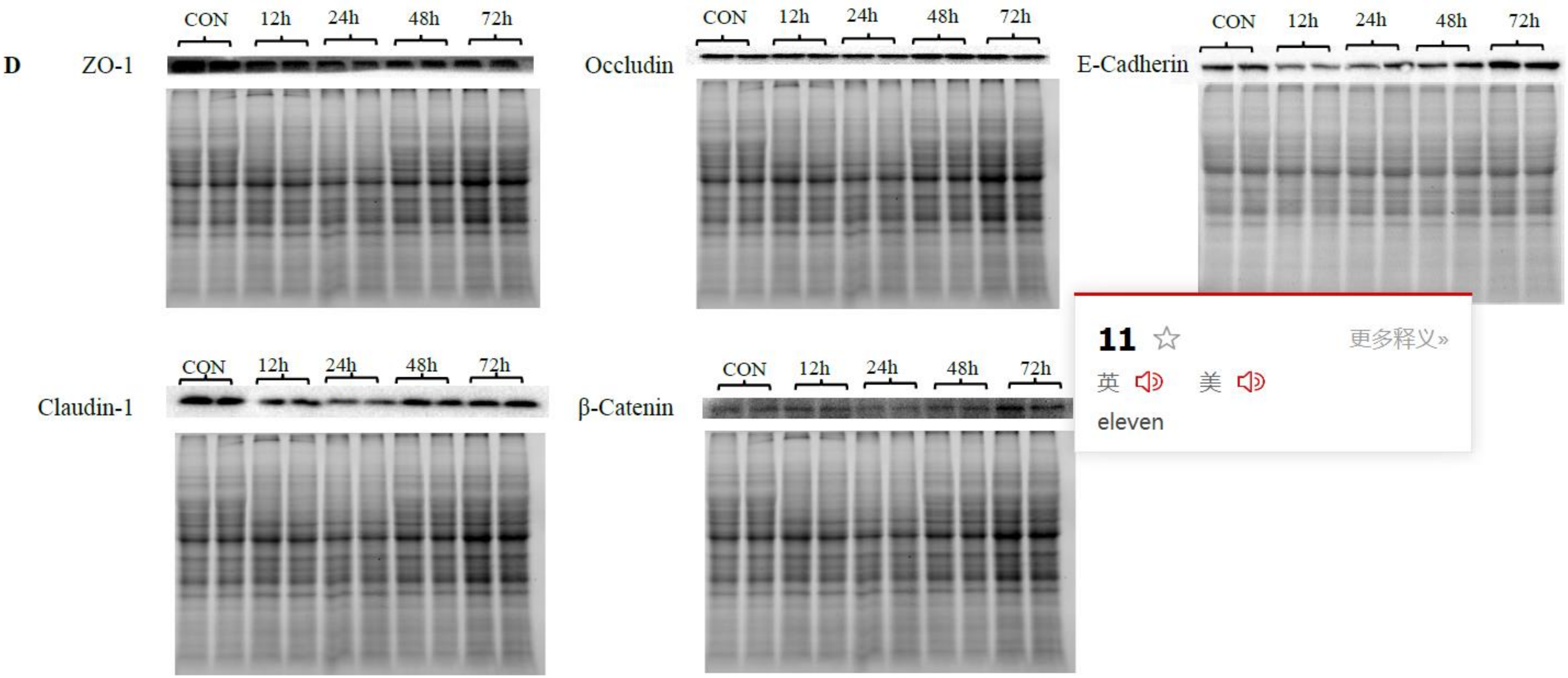

11 ☆ 更多释义»
英 美
eleven

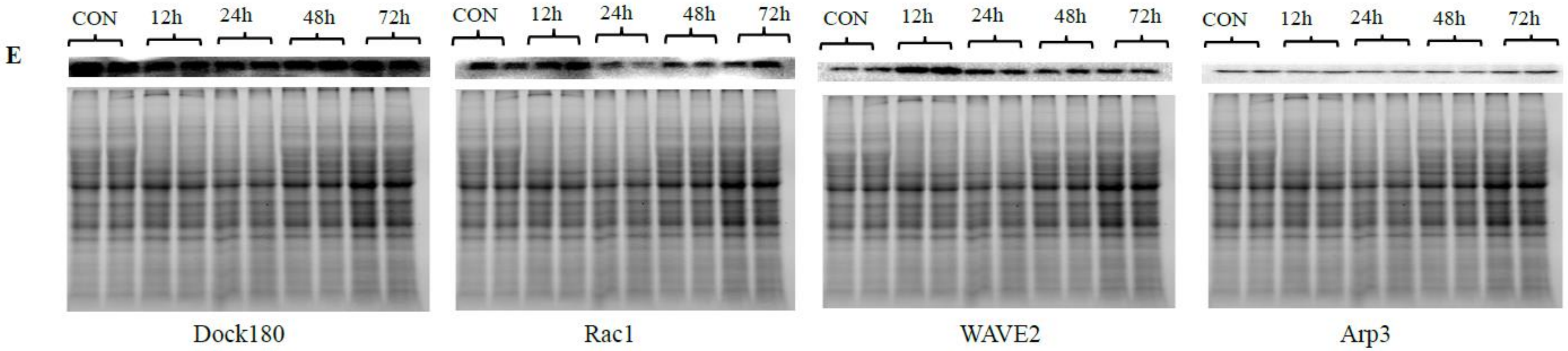

Figure S6. Expression of TJ and AJ proteins in Caco-2 cells with different duration of microgravity simulation (**A**). Several proteins were down-regulated under 48 h-SMG. TEM images of Caco-2 cells with different duration of microgravity simulation (**B**). The results showed that 48 h-SMG led to most serious damage with broken microvilli and enlarged cellular space. Expression of Racl-WAVE2-Arp3 pathway proteins in Caco-2 cells with different duration of microgravity simulation (**C**). All proteins except Dock180 were down-regulated under 48 h-SMG. Note: * $p < 0.05$ and ** $p < 0.01$, compared with CON group. The relative expression levels of proteins were expressed as the ratio of gray value of the target band to that of total proteins in the same sample. The total proteins of AJ, TJ, Racl-WAVE 2-Arp3 pathway proteins in Caco-2 cells with different duration of microgravity simulation (**D-E**).

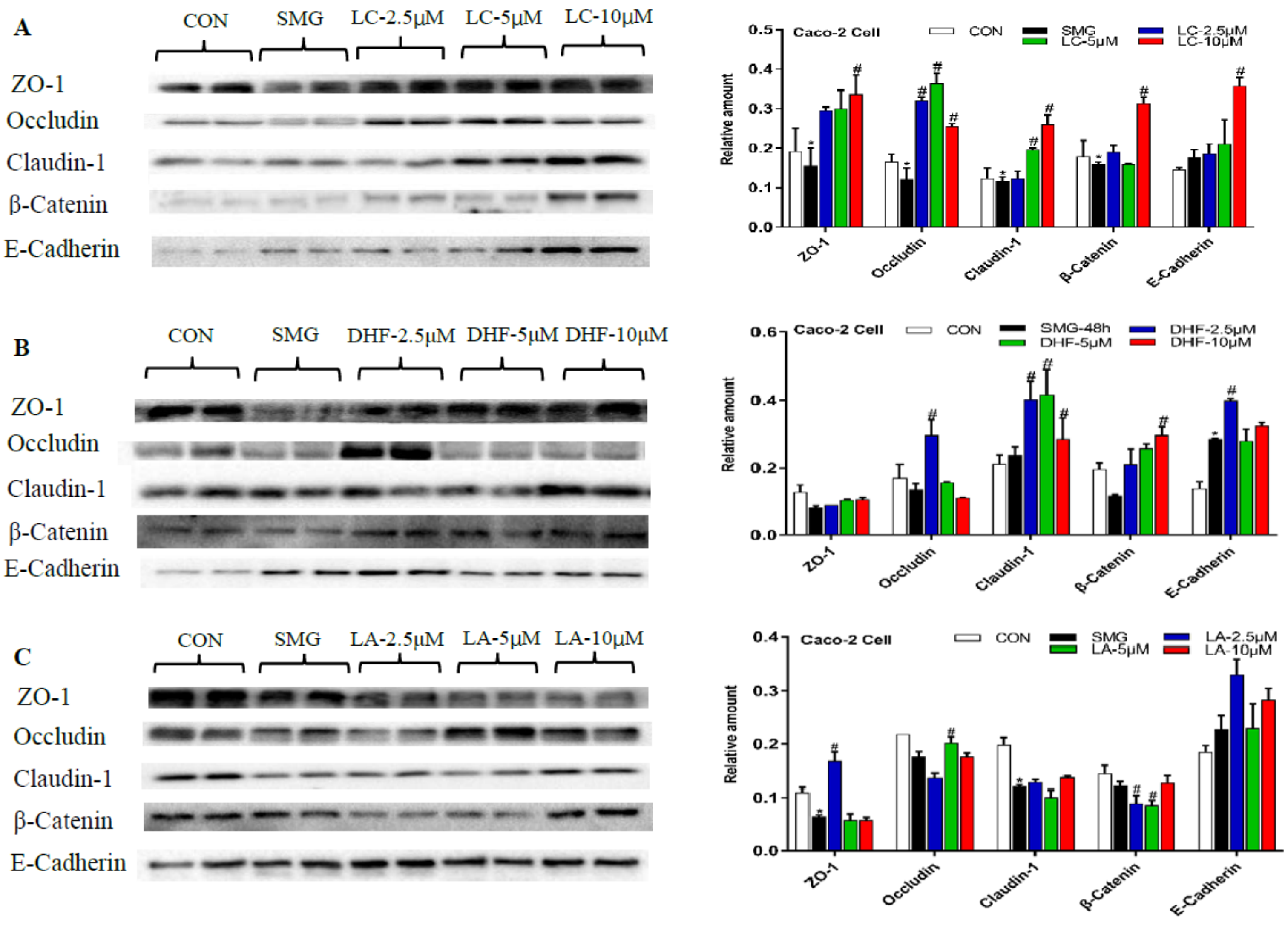
A
CON
SMG
LC-2.5μM
LC-5μM
LC-10μM
ZO-1
Occludin
Claudin-1
β-Catenin
E-Cadherin
Caco-2 Cell
CON
SMG
LC-2.5μM
LC-5μM
LC-10μM
Relative amount
B
CON
SMG
DHF-2.5μM
DHF-5μM
DHF-10μM
ZO-1
Occludin
Claudin-1
β-Catenin
E-Cadherin
Caco-2 Cell
CON
SMG-48h
DHF-2.5μM
DHF-5μM
DHF-10μM
Relative amount
C
CON
SMG
LA-2.5μM
LA-5μM
LA-10μM
ZO-1
Occludin
Claudin-1
β-Catenin
E-Cadherin
Caco-2 Cell
CON
SMG
LA-2.5μM
LA-5μM
LA-10μM
Relative amount

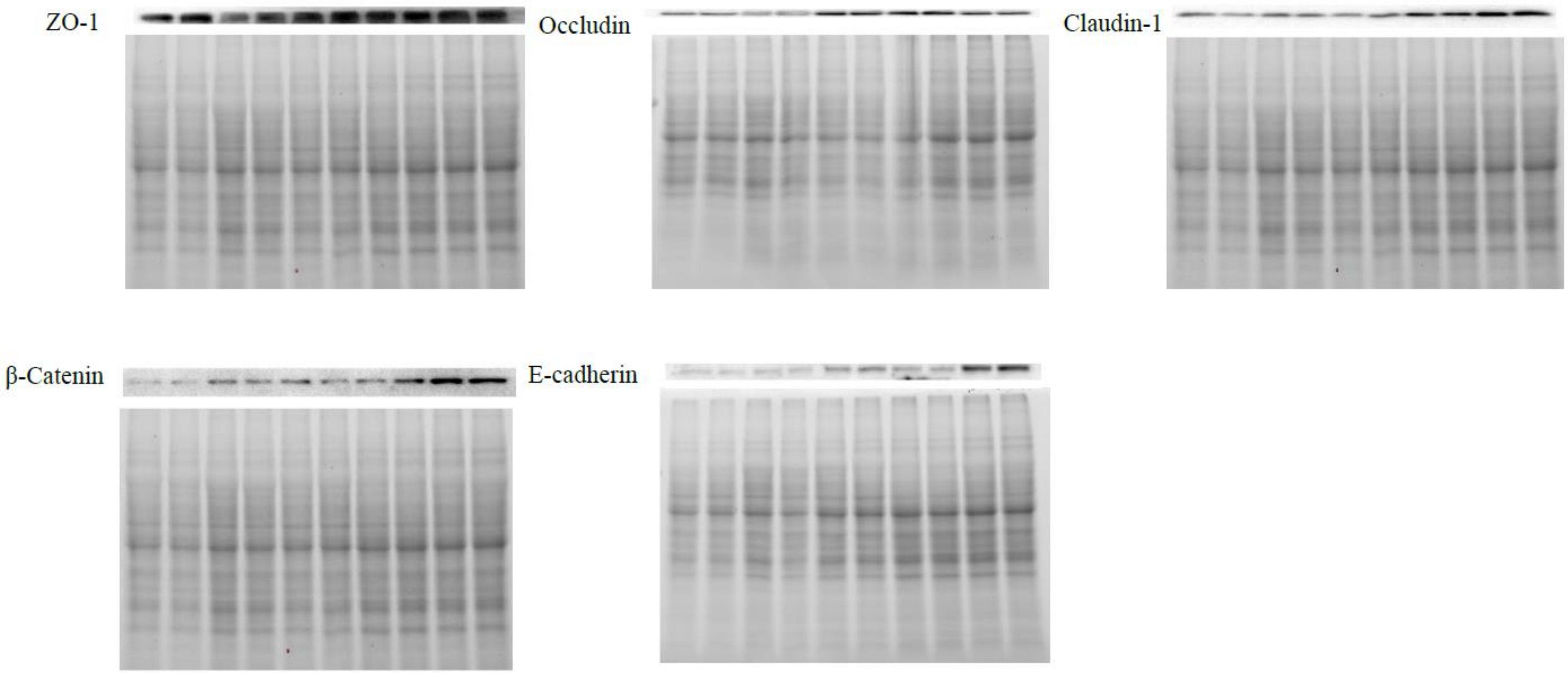
ZO-1
Occludin
Claudin-1
β-Catenin
E-cadherin

D-LC

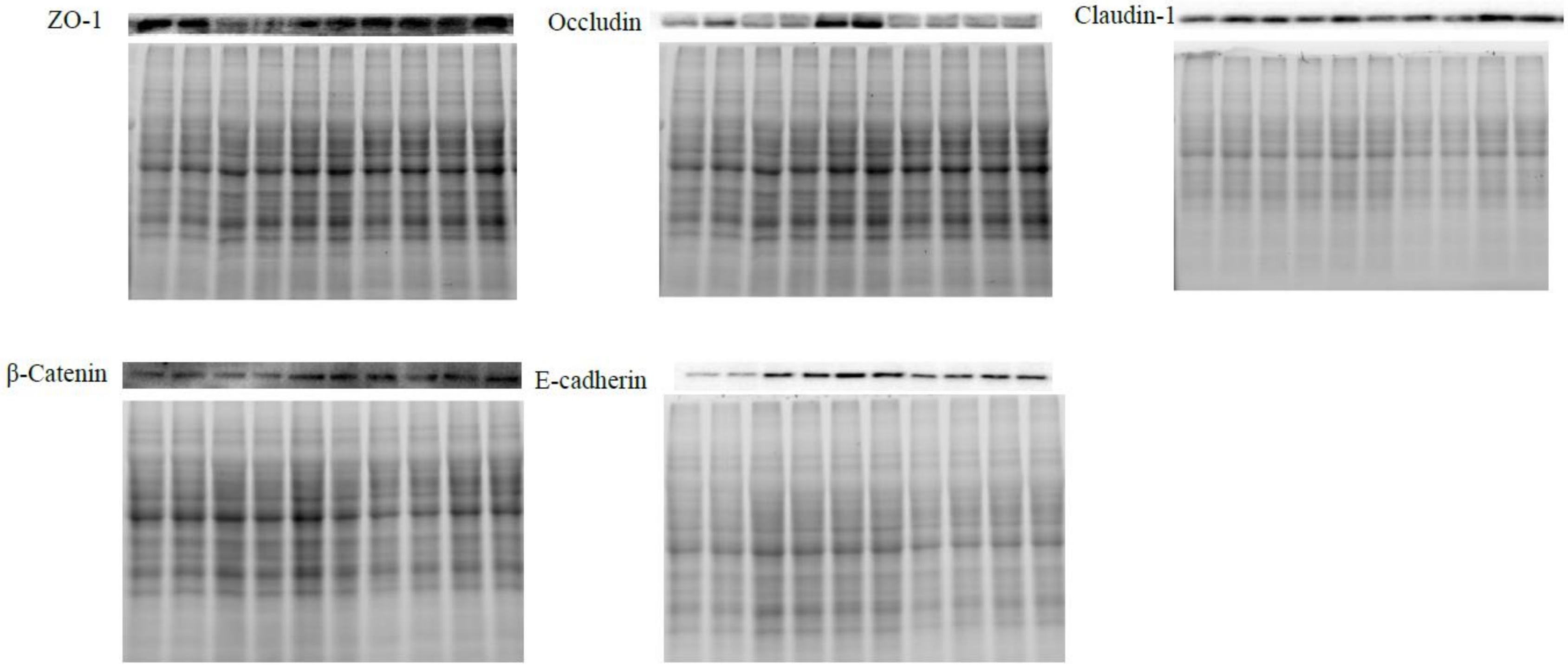

E-7,4´-DHF

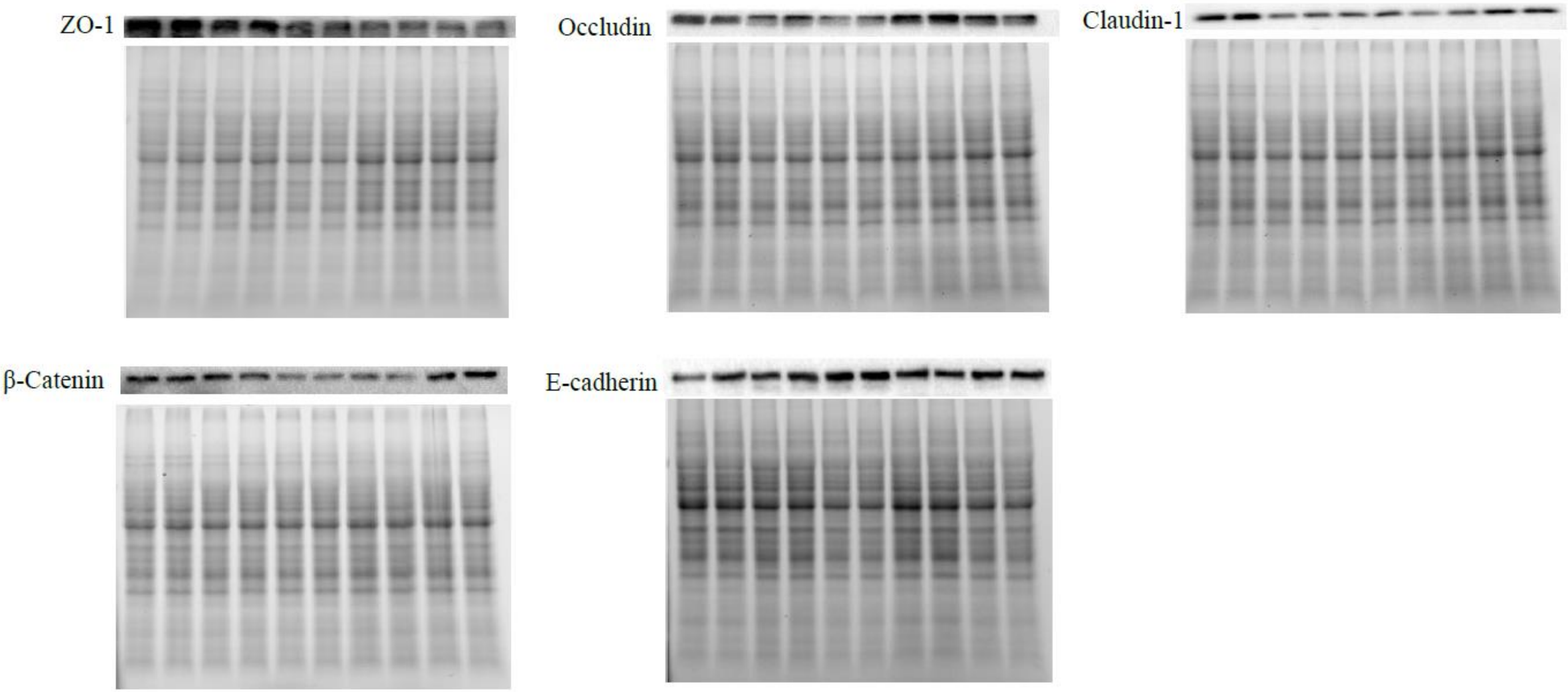

F-LA

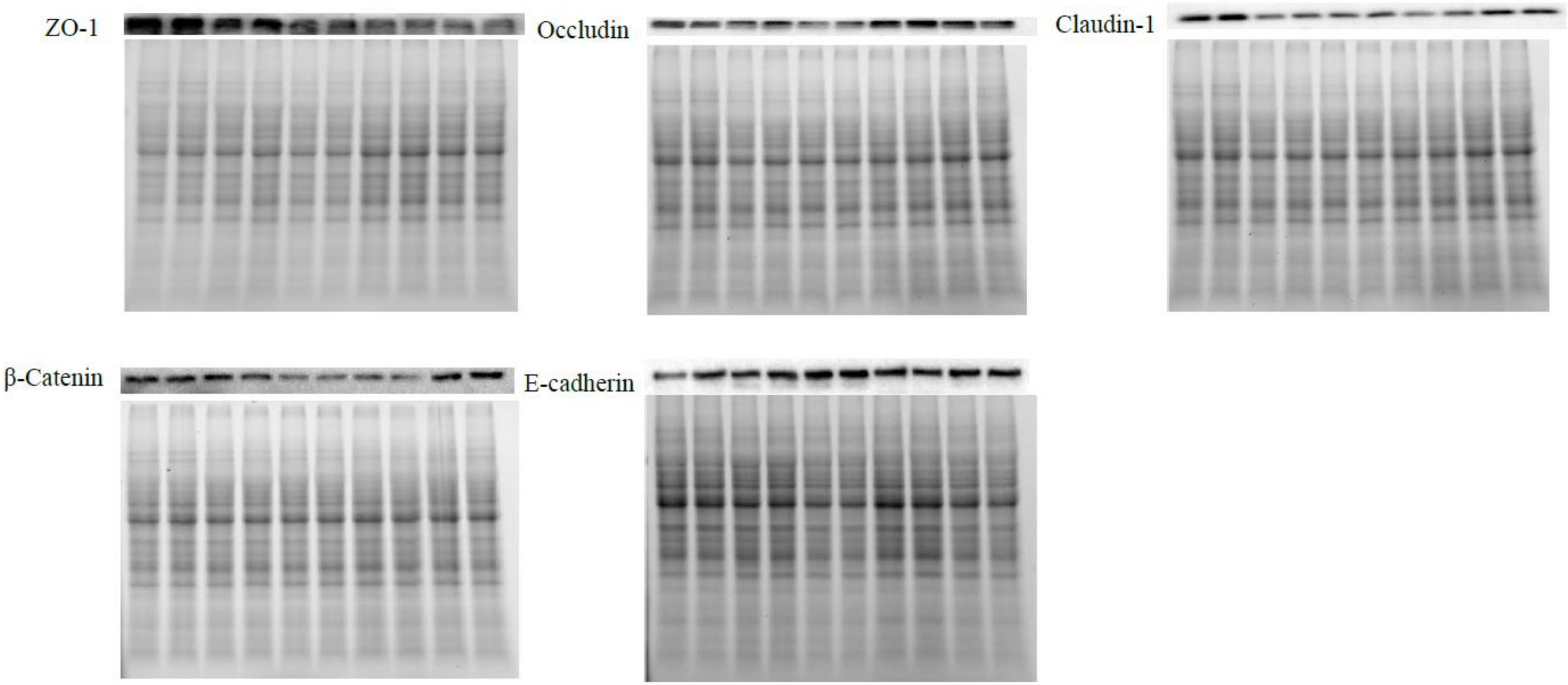

G-LC

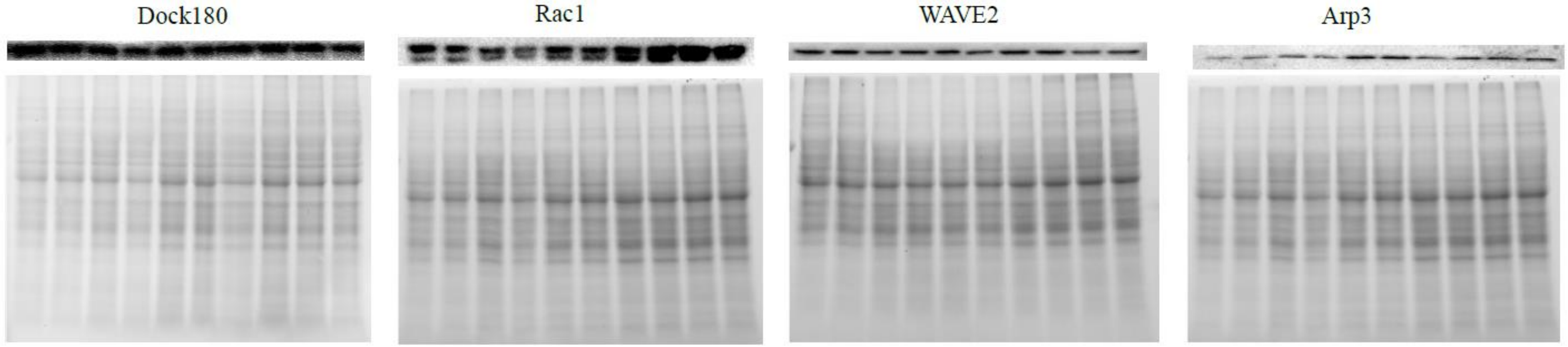

H-7,4´-DHF

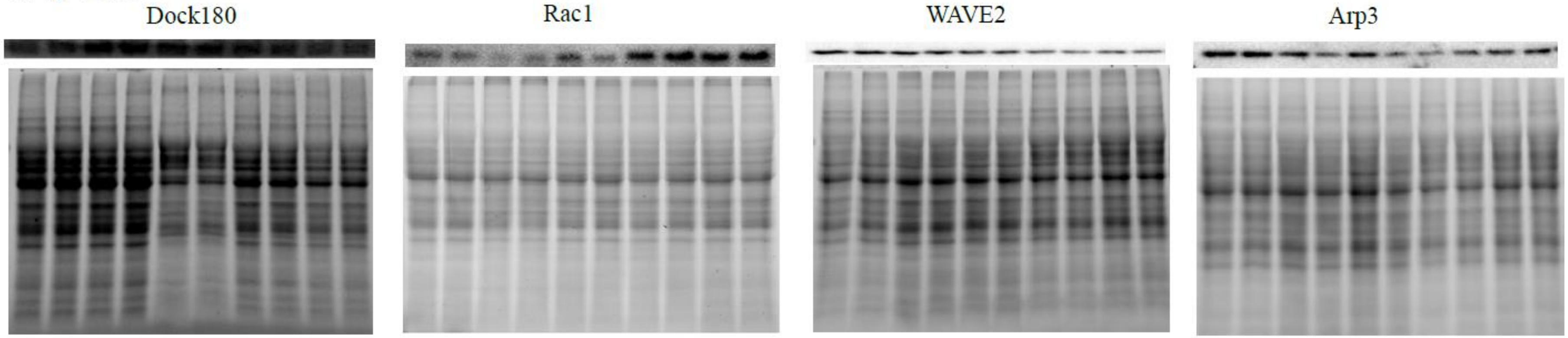

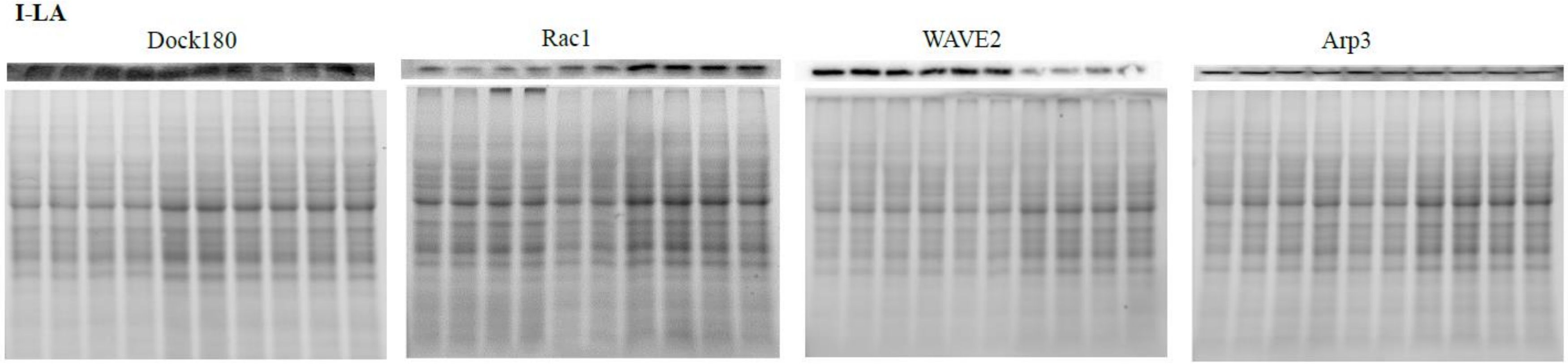

Figure S7. Expression of AJ and TJ proteins in SMG-treated Caco-2 cells incubated with LC (**A**), 7,4′-DHF (**B**) and LA (**C**). LC and LA increased AJ and TJ proteins better than 7,4′-DHF. The total proteins gel corresponding to every band was listed in **D** (LC), **E** (7,4′-DHF), and **F** (LA), respectively. The Western-blot bands of Racl-WAVE2-Arp3 pathway proteins in SMG-treated Caco-2 and their corresponding total proteins gel of LC (**G**), 7,4′-DHF (**H**) and LA (**I**).

Supplementary Table S1 Docking energy (Kcal/mol) of different differentially expressed proteins and chemical structures of loureirin A, loureirin B, loureirin C, 7,4'-DHF, pterostilbene and resveratrol.

Num	Gene Name	Protein ID	Docking energy (Kcal/mol)					
			Loureirin A	Loureirin B	Loureirin C	7,4'- DHF	Resveratrol	Pterostilbene
1	actin, gamma 1(Actg1)	P63259	-7.24	-6.81	-6.59	-6.54	-6.21	-5.34
2	phosphoinositide-3-kinase regulatory subunit 1(Pik3r1)	F1LNG5	-7.15	-6.2	-6.51	-6.17	-6.03	-5.72
3	integrin subunit beta 1(Itgb1)	A0A0G2JSK5	-5.52	-5.38	-5.46	-6.71	-5.94	-6.68
4	actinin, alpha 1(Actn1)	Q6T487	-6.15	-6.24	-6.12	-6.5	-6.18	-6.8
5	vinculin (Vcl)	R9PXU6	-7.61	-7.24	-7.01	-7.02	-6.84	-6.73
6	rho-associated coiled-coil containing protein kinase 2(Rock2)	F1LQT3	-6.56	-6.58	-7.16	-7.02	-5.99	-5.5
7	protein phosphatase 1, regulatory subunit 12A(Ppp1r12a)	A0A0G2JWJ0	-6.59	-5.69	-5.88	-7.52	-6.87	-6.89
8	myosin light chain kinase(Mylk)	A0A0G2K0Q7	-7.61	-6.08	-6.5	-9.31	-8.52	-8.34
9	integrin alpha 2(Itga2)	A0A0G2K470	-5.92	-5.9	-6.01	-6.95	-6.59	-6.07
10	rho-associated coiled-coil containing protein kinase 1(Rock1)	D3ZN37	-6.24	-5.76	-5.86	-6.97	-6.17	-6.15
11	dedicator of cyto-kinesis 1(Dock1)	D3ZZW1	-6.08	-5.64	-7.27	-6.27	-6.46	-6.61
12	mitogen activated protein kinase kinase 1(Map2k1)	Q01986	-7.07	-6.9	-8.67	-7.29	-7.6	-7.62
13	v-crk avian sarcoma virus CT10 oncogene homolog-like(Crkl)	Q5U2U2	-6.62	-6.69	-7.57	-6.41	-6.93	-6.97
14	p21 (RAC1) activated kinase 4(Pak4)	B5DF62	-6.34	-5.79	-6.09	-6.58	-6.19	-6.43
15	integrin subunit beta 4(Itgb4)	F1LSD3	-6.03	-5.99	-5.5	-6.28	-6.07	-6.71
16	integrin subunit alpha 6(Itga6)	G3V667	-7.47	-7.34	-7.69	-6.06	-5.89	-5.33
17	p21 (RAC1) activated kinase 1(Pak1)	P35465	-6.3	-5.66	-6.04	-6.71	-6.29	-6.36
18	integrin subunit beta 6(Itgb6)	Q6AYF4	-6.53	-5.8	-6.59	-8.09	-6.88	-6.99
19	IQ motif containing GTPase activating protein 1(Iqgap1)	G3V7Q7	-5.76	-5.33	-5.61	-6.63	-6.04	-6.28
20	mitogen activated protein kinase kinase 2(Map2k2)	P36506	-6.73	-5.96	-7.81	-7.16	-7.69	-7.52
21	cytoplasmic FMR1 interacting protein 1(Cyfip1)	A0A0G2K472	-7.37	-6.52	-6.79	-7.83	-7.95	-7.09

Continued

22	IQ motif containing GTPase activating protein 2(Iqgap2)	F1LW74	-6.28	-6.25	-6.11	-7.18	-5.96	-6.29
23	phosphatidylinositol-5-phosphate 4-kinase type 2 gamma(Pip4k2c)	O88370	-6.29	-5.62	-6.85	-6.98	-7.07	-6.81
24	ezrin(Ezr)	P31977	-5.52	-5.05	-5.47	-6.18	-5.8	-6.05
25	NCK-associated protein 1(Nckap1)	P55161	-6.03	-5.61	-5.99	-6.83	-6.02	-6.29
26	erb-b2 receptor tyrosine kinase 2(Erbb2)	A0A0G2JT61	-7.5	-7.11	-6.72	-6.75	-6.91	-6.45
27	filamin B(Flnb)	D4A8D5	-6.05	-5.35	-5.78	-6.68	-5.71	-6.1
28	laminin subunit beta 3(Lamb3)	F1LPI5	-7.87	-7.44	-7.39	-6.75	-6.61	-5.87
29	talin 1(Tln1)	G3V852	-5.92	-6	-6.36	-6.99	-6.28	-6.09
30	zyxin(Zyx)	D4A7U1	-6.28	-5.59	-5.77	-6.62	-5.8	-6.02
31	protein kinase C, alpha(Prkca)	F1M2P8	-6.59	-6.56	-6.62	-7.21	-6.5	-6.54
32	tight junction protein 1(Tjp1)	A0A0G2K2P5	-8.1	-7.5	-7.31	-7.04	-7.01	-7.11
33	cortactin(Cttn)	Q66HL2	-7.11	-6.78	-6.7	-6.31	-5.63	-5.33
34	claudin 2(Cldn2)	A0A0G2JYB2	-6.11	-6.21	-6.11	-6.31	-5.71	-5.96
35	tight junction protein 3(Tjp3)	A0A0G2K8M3	-6.49	-6.48	-6.3	-7.32	-6.47	-6.59
36	myosin heavy chain 14(Myh14)	F1LNF0	-7.22	-6.64	-6.62	-8.07	-7.61	-7.86
37	symplekin(Sympk)	F1LSH0	-6.55	-5.71	-6.54	-7.03	-6.34	-7.06
38	myosin, heavy chain 9, non-muscle-like 1(Myh9l1)	G3V6P7	-6.61	-6.11	-7.09	-7.06	-6.52	-6.79
39	tight junction protein 2(Tjp2)	Q3ZB99	-6.55	-6	-6.86	-7.27	-6.45	-6.53
40	protein phosphatase 2 scaffold subunit A beta(Ppp2r1b)	Q4QQT4	-5.35	-5.14	-5.82	-6.25	-6.19	-6.24
41	protein phosphatase 2 scaffold subunit A alpha(Ppp2r1a)	Q5XI34	-6.21	-6.03	-6.3	-6.24	-5.64	-6.01
42	claudin 3(Cldn3)	Q63400	-6.25	-5.98	-6.24	-7.04	-6.49	-6.44
43	catenin alpha 1(Ctnna1)	Q5U302	-6.61	-6.5	-6.66	-7.92	-7.49	-7.46
44	dynamin 2(Dnm2)	A0A0A0MY48	-7.87	-5.78	-6.95	-8.23	-7.44	-7.79
45	engulfment and cell motility 1(Elmo1)	A0A0G2K4S6	-5.37	-4.55	-5.27	-6.13	-5.37	-5.56

Continued

46	CD2-associated protein(Cd2ap)	F1LRS8	-6.32	-5.58	-5.94	-7.14	-6.96	-6.82
47	clathrin heavy chain(Cltc)	F1M779	-7.15	-7.05	-7.33	-7.17	-6.97	-6.96
48	engulfment and cell motility 3(Elmo3)	Q499U2	-6.06	-5.93	-6.04	-7.28	-6.65	-7.1
49	SMAD family member 4(Smad4)	A0A0G2JXW2	-6.17	-5.65	-6.49	-6.86	-6.5	-6.1
50	catenin delta 1(Ctnnd1)	D3ZZZ9	-6.11	-5.49	-6.91	-6.3	-6.29	-6.39
51	nectin cell adhesion molecule 3(Nectin3)	D4A5C0	-5.33	-5.09	-5.73	-6.21	-5.33	-5.42
52	protein tyrosine phosphatase, receptor type, F(Ptprf)	G3V8P4	-5.97	-5.05	-5.28	-6.72	-6.13	-6.07
53	Ras-related C3 botulinum toxin substrate 1 (Rac1)	Q6RUV5	-5.88	-5.88	-7.04	-6.15	-6.65	-6.12
54	Actin-related protein 3 (Arp3)	Q4V7C7	-8.09	-8.09	-8.35	-7.65	-7.83	-6.49
55	β-catenin (Ctnnb)	Q9WU82	-5.17	-4.63	-5.2	-6.32	-5.63	-5.57
56	Occludin (Ocln)	Q6P6T5	-4.26	-3.87	-4.02	-5.21	-4.58	-4.73
57	Claudin-1 (Cldn)	P56745	-5.82	-5.57	-5.39	-6.66	-5.69	-5.89
58	E-Cadherin (Cdh1)	Q9R0T4	-7.38	-7.14	-7.71	-6.34	-6.74	-5.46

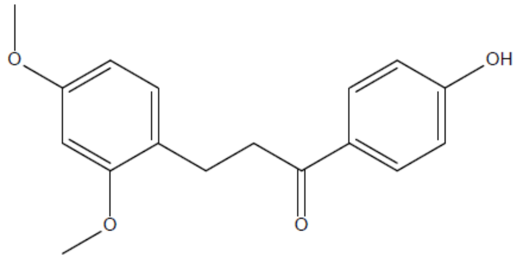

Loureirin A

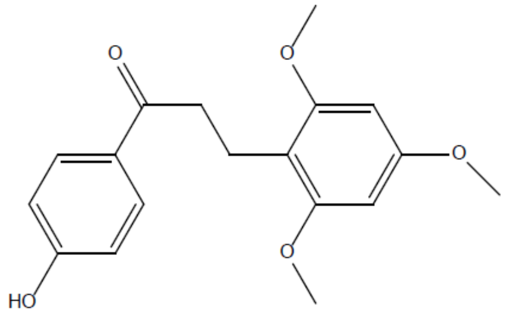

Loureirin B

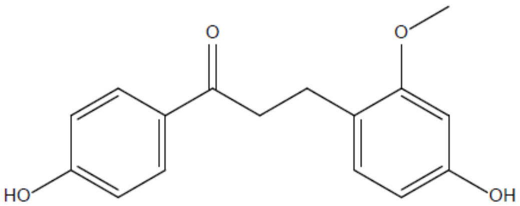

Loureirin C

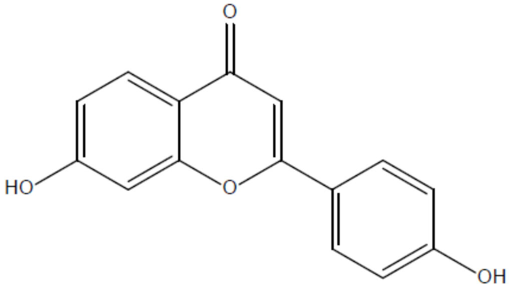

7,4'-Dihydroxyflavone

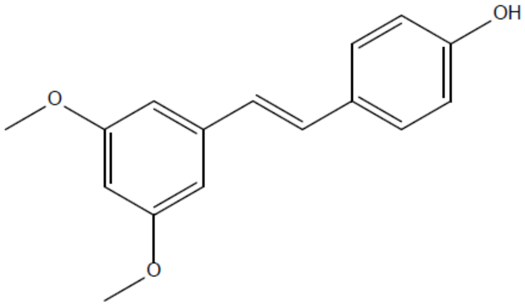

Pterostilbene

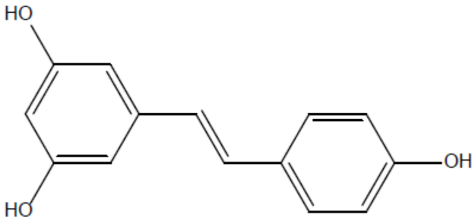

Resveratrol
